# Exploring the potential of *Lactocaseibacillus rhamnosus* PMC203 in inducing autophagy to reduce the burden of *Mycobacterium tuberculosis*

**DOI:** 10.1007/s00430-024-00794-z

**Published:** 2024-07-09

**Authors:** Md Abdur Rahim, Hoonhee Seo, Sukyung Kim, Indrajeet Barman, Fatemeh Ghorbanian, Mohammed Solayman Hossain, Md Sarower Hossen Shuvo, Saebim Lee, Ho-Yeon Song

**Affiliations:** 1 Department of Microbiology and Immunology, School of Medicine, Soonchunhyang University, Cheonan, 31151 Republic of Korea; 2 Human Microbiome Medical Research Center, Soonchunhyang University, Asan, 31538 Republic of Korea

**Keywords:** Probiotics, Anti-tuberculosis, Autophagy, *Lactocaseibacillus rhamnosus* PMC203, *Mycobacterium tuberculosis*

## Abstract

*Mycobacterium tuberculosis*, a lethal pathogen in human history, causes millions of deaths annually, which demands the development of new concepts of drugs. Considering this fact, earlier research has explored the anti-tuberculosis potential of a probiotic strain, *Lactocaseibacillus rhamnosus* PMC203, leading to a subsequent focus on the molecular mechanism involved in its effect, particularly on autophagy. In this current study, immunoblotting-based assay exhibited a remarkable expression of autophagy marker LC3-II in the PMC203 treated group compared to an untreated group. A remarkable degradation of p62 was also noticed within treated cells compared to control. Furthermore, the immunofluorescence-based assay showed significant fold change in fluorescence intensity for alexa-647-LC3 and alexa-488-LC3, whereas p62 was degraded noticeably. Moreover, lysosomal biogenesis generation was elevated significantly in terms of LAMP1 and acidic vesicular organelles. As a result, PMC203-induced autophagy played a vital role in reducing *M. tuberculosis* burden within the macrophages in treated groups compared to untreated group. A colony -forming unit assay also revealed a significant reduction in *M. tuberculosis* in the treated cells over time. Additionally, the candidate strain significantly upregulated the expression of autophagy induction and lysosomal biogenesis genes. Together, these results could enrich our current knowledge of probiotics-mediated autophagy in tuberculosis and suggest its implications for innovatively managing tuberculosis.

**Supplementary Information:**

The online version contains supplementary material available at 10.1007/s00430-024-00794-z.

## Introduction

Tuberculosis (TB) caused by *Mycobacterium tuberculosis*, an ancient pathogen in humans, remains a global health emergency [1]. In 2021, 10.6 million newly active tuberculosis cases were identified, which showed an increase of 4.5% from the previous year, according to the World Health Organization (WHO) reports for 2022 [2]. Additionally, 1.6 million individuals died in 2021 due to this lethal pathogen, whereas 1.5 and 1.4 million deaths were recorded in 2020 and 2019, respectively, indicating a growing severity of this disease [2]. Moreover, the prevalence of drug-resistant tuberculosis increased from 2020 to 2021, with a total of 450,000 new cases estimated in 2021 despite necessary initiatives being taken to reduce its global incidence [2]. Furthermore, therapies used for tuberculosis treatment for more than four decades show toxicities and require prolonged treatment regimens [3]. Hence, developing an alternative anti-TB agent with a novel mechanism with enhanced efficacy and safety is urgently necessary [4]. Considering the circumstances above, various approaches are being explored to combat this lethal pathogen [5]. In this context, probiotics have emerged as one of the innovative ways of treating tuberculosis [6] due to their established effectiveness against various infectious diseases [7].

According to the WHO, probiotics are living microorganisms that can provide health benefits to the host when consumed in appropriate quantities [8]. They are increasingly employed to treat a wide variety of disorders or diseases due to their established benefits with fewer adverse effects [9], including hypocholesterolemia, gastrointestinal disorder, ulcerative colitis, Crohn’s diseases, diabetes, atopy, allergy, and psychological stress [10–14]. Probiotics also show excellent effectiveness against various infectious diseases caused by pathogens, including superbugs [15–19]. Following this trend, probiotics have recently been applied to treat tuberculosis, although their use is in its early stages regarding effectiveness and action mechanisms [20]. However, the way probiotics exhibit their advantageous impact has been investigated, and autophagy has been identified as one of the principal mechanisms initiating beneficial outcomes [21].

Autophagy is an evolutionarily maintained cellular process in which a double-membraned organelle called autophagosome sequesters cytoplasmic targets and fuses with lysosomes, resulting in the formation of autolysosomes that undergo degradation through the action of lysosomal enzymes [22]. This dynamic process plays various physiological and pathophysiological functions [23]. Regarding this context, autophagy can be induced by probiotics. Thus, this induced autophagy has been used to treat various diseases and disorders, including metabolic disorders, cardiovascular diseases, neurodegenerative disorders, liver injury, and cancer [24–28]. Thus, autophagy has emerged as a new and potent way to treat various diseases. Beyond these functions, probiotics-mediated autophagy has shown excellent efficacy against pathogenic microorganisms such as *Salmonella enterica* and *Escherichia coli* [29, 30]. Similarly, there are studies that have reported autophagy as a defense mechanism against *M. tuberculosis* both in in vitro and in vivo level [31–33]. A separate investigation has also revealed the protective response of autophagy against *M. tuberculosis*, emphasizing the importance of the autophagic process in responses to lethal pathogens [33]. Considering these facts, several autophagy-based TB therapies have recently been explored, demonstrating high potential for future clinical trials [34]. However, since it is at an early level, comprehensive and in-depth research is needed to develop a promising and potentially effective autophagy-based anti-TB therapeutic agent.

Our previous study identified numerous strains from various sources, from food to the human body, to find the potential probiotic strain [35]. Subsequently, in the rigorous anti-tuberculosis screening process, vaginal microbiota-derived *Lactocaseibacillus rhamnosus* PMC203 showed a remarkable intracellular impact on both drug-sensitive and drug-resistant *M. tuberculosis* strains. In addition, it demonstrated its capacity to suppress the growth of this lethal pathogen in the anti-mycobacterial susceptibility assays tested. In that study, we also observed the autophagy induction by PMC203 but did not extensively explore the molecular mechanism. Therefore, our present study mainly focused on the PMC203-stimulated autophagy and its subsequent contribution to the clearance of *M. tuberculosis* burdens in macrophage cells.

## Materials and methods

### Cell line and bacterial strains

A murine macrophage cell line RAW264.7 (KCLB 40071) was obtained from the Korean Cell Line Bank and cultured in Dulbecco's Modified Eagle Medium (DMEM, Gibco, USA) using a humidified CO_2_ incubator at 37 °C. The DMEM was supplemented with 10% fetal bovine serum (FBS) and 1% antibiotics (HyClone, USA). For all experiments, mycoplasma-free cells were initially seeded at 1 × 10^5^ cells/ml concentration and used until they reached a confluence level of 75–85%.

The probiotic strain *Lacticaseibacillus rhamnosus* PMC203 identified from vaginal microbiota that was discussed in detail in our previous publications [35] was cultivated in MRS broth at 37 °C. Following an overnight incubation, the grown culture was centrifuged, washed, and suspended in 1 × PBS. The obtained bacteria were then exposed to a heat treatment at 100 °C for 30 min [36]. Afterward, heat killed bacteria precipitation was collected through centrifugation and resuspended in DMEM for cell treatment. This prepared final resuspension was also spread onto the MRS agar plate to ensure they are dead.

*M. tuberculosis* strain H37Rv (ATCC 27294) was acquired from the American Type Culture Collection (ATCC, USA). A recombinant strain of *M. tuberculosis* (H37Ra-GFP) previously prepared in our laboratory was also used [37]. All bacterial strains were cultivated in Middlebrook 7H9 broth or Middlebrook 7H10 agar (BD Difco, USA) containing 0.5% Tween 80 (Sigma-Aldrich, USA) and 10% OADC (oleic acid-albumin-dextrose-catalase) (BD Difco). However, all related experiments with *M. tuberculosis* were performed in an Animal Biosafety Level 3 Laboratory (ABSL-3, KDCA-20-3-04) at Soonchunhyang University in compliance with biosafety regulations.

### Cell cytotoxicity assay

The impact of PMC203 on RAW264.7 cells was assessed using a cell viability assay kit (DoGenBio, Korea) with water-soluble tetrazolium salt (WST). Overnight-grown cells were treated with the probiotic strain ranging from 1 × 10^5^ to 1 × 10^9^ CFU/ml for 24 h. Afterward, cell cytotoxicity was determined at 570 nm employing a multiplate reader (Perkin Elmer, USA). In addition, viable cells after PMC203 treatment were counted with a hemocytometer using trypan blue (Gibco, USA). Moreover, methylene blue stain (Dagatron, Korea) was used to observe morphological changes in treated cells.

### Western blot of autophagy-related proteins LC3 and p62

Autophagy induction of PMC203 was explored using Western blot technology. Confluent macrophage monolayer cells were treated with PMC203, incubated for different periods, and then proteins were extracted using 1 × RIPA lysis buffer. In the case of chloroquine (Cq) (25 µM)/ infection treatment, cells were first subjected to Cq or H37Rv (multiplicity of infection = 30:1) treatment for 2 h and then incubated alone or with PMC203 for 6 h. Extracted proteins were then quantified, transferred into polyvinylidene difluoride membranes, and incubated with primary antibodies including LC3 (Sigma, USA), p62 (Cell Signal Technologies, USA), mTOR (Cell Signal Technologies), Beclin1 (Cell Signal Technologies), and β-actin (Cell Signal Technologies) followed by incubation with horseradish peroxidase‑conjugated secondary antibody (Cell Signal Technologies). Membranes were then visualized employing a chemidoc XRS system (Bio-Rad, USA), and densitometric measurement of obtained bands was subsequently conducted with Image J software (version 1.8.0).

### Immunofluorescence assay for detection of autophagy-specific proteins

Immunofluorescence staining was performed to detect autophagy-related proteins. RAW264.7 cells were cultured on coverslips and treated with PMC203 or rapamycin (Rapa) for 6 h. In the infection assay, cells were treated with GFP-H37Ra at a multiplicity of infection of 30:1 for 2 h and then treated with PMC203 for 24 h. Cells were then fixed, permeabilized, and blocked using paraformaldehyde, Triton X-100, and bovine serum albumin, respectively. Next, primary antibodies, including non-conjugated LC3, LC3 conjugated with Alexa flour 488 (Novusbio, USA), p62 (Medchem express, USA), and LAMP1 (Abcam, UK), were applied to the cells, followed by overnight incubation at 4 °C. Afterward, cells were treated with a secondary antibody conjugated with Alexa Fluor 647, if primary antibodies were not conjugated. Next, they were washed and counterstained with DAPI (Abcam, UK) for 10 min, mounted with an anti-fade fluorescence mounting medium (Abcam), and finally imaged with a laser-scanning confocal microscope (ZEISS 800 LSM, Germany).

### Labelling of autophagic vacuoles

The autophagic flux induced by PMC203 was assessed using an autophagy detection kit (Abcam, USA) following the manufacturer’s instructions. Cells were cultured onto coverslips, treated with PMC203 or Rapa (50 µM), and incubated for 6 h. After the cells were washed with 1 × assay buffer, 100 µl of dual detection of reagent (prepared using green detection dye and nuclear stain) was added to cover the monolayer cell. After 30 min of incubation at 37 °C, cells were carefully washed and observed using a confocal microscope with standard FITC (green) and DAPI (blue) filter sets. Obtained images were then analyzed using ZEN software (version 3.7.97.01000).

### Formation of acidic vesicular organelles

The generation of acidic vesicular organelles (AVOs) after PMC203 treatment was investigated using acridine orange dye (Invitrogen, USA). Overnight grown confluent monolayer of cells was subjected to probiotic or Rapa treatment for 6 h. After washing with PBS, 5 µg/ml of acridine orange dye was added to cells and further incubated at 37 °C for 30 min. Next, cells were washed and imaged using a laser-scanning confocal microscope.

### Quantification of the lysosomal marker LAMP1

Immunofluorescence staining was performed to detect the expression of a lysosomal marker. RAW264.7 cells were cultured on coverslips and treated with PMC203 or Rapa for 6 h. Subsequently, staining was conducted as described in the earlier section. In this assay, the LAMP1 (Abcam, UK) primary antibody was diluted following the manufacturer’s instructions. Following primary antibody treatment, cells were incubated with secondary antibody and subsequently imaged with a laser-scanning confocal microscope.

### CFU-based PMC203 mediated anti-tuberculosis assay

An antibacterial assay was performed to assess the impact of PMC203-mediated autophagy on reducing the burden of *M. tuberculosis*. Cultured cells were first exposed to H37Rv for 2 h. After incubation, they were washed three times with 1 × PBS to remove extracellular H37Rv and treated with PMC203 and/or Cq/3-methyladenine (3-MA) (Sigma, USA). For phagocytosis analysis, cells were initially treated with Cq/3-MA for 2 h, followed by PMC203 treatment for 6 h. They were then exposed to *M. tuberculosis*. After 0, 24, and 72 h of incubation, cells were lysed, and cell lysates were serially diluted. Finally, diluted lysates were placed onto H710 agar plates for quantifying viable bacteria. At 0 h of incubation, AFB staining was also conducted to ensure the engulfment of *M. tuberculosis* in a similar pattern across all groups.

### Transfection of RAW264.7 cells with small interference RNA

Macrophage RAW264.7 cells were transfected with ATG5 siRNA at the final concentration of 50 nM (Invitrogen, USA) using lipofectamine 2000 (Invitrogen, USA) following the manufacturer’s instructions. After 48 h of incubation, knockdown efficacy was checked. Simultaneously, transfected cells were treated with H37Rv and/or PMC203, and then immunofluorescence was conducted. At the same time, acid-fast bacilli (AFB) staining was also performed following the previously published method [38]. The experiments used nonspecific siRNA (Invitrogen, USA) as a negative control.

### Quantitative real-time PCR analysis

The autophagy induction and lysosomal biogenesis gene expression pattern in RAW 264.7 cells were explored after H37Rv and/or PMC203 treatment. The confluent monolayer of macrophages was treated with H37Rv and then incubated with or without PMC203 for 24 h. Afterward, total RNA was extracted employing an RNA extraction kit (Qiagen, Germany), and subsequently, cDNA was transcribed following the manufacturer’s protocol. RT-PCR was then performed with a SYBR green reagent employing a Real-Time PCR system (Applied Biosystems, USA), and the resulting data was subsequently analyzed. Primers for induction genes [39, 40] and lysosomal biogenesis genes [41, 42] used in this assay are listed in Supplementary Table S1.

### Measurement of reactive oxygen species

Reactive oxygen species (ROS) generation in macrophages was evaluated using a ROS-Glo H_2_O_2_ Assay kit (Promega, USA). Confluent monolayer cells were exposed to H37Rv for 2 h and treated with PMC203 for 24 h. Afterward, an H_2_O_2_ substrate solution was prepared and applied to all cells, followed by incubation for 6 h. Subsequently, ROS Glo reagent was added to cells and incubated for 20 min, and the ROS level was measured using a multiplate reader.

### Enzymatic activity of PMC203

The enzyme-producing capacity of the probiotic strain was investigated utilizing an API ZYM kit (BioMerieux, France). PMC203 was first grown in MRS broth and adjusted to 5 McFarland standard. After that, 75 µl of cell suspension was added to each well of the stipe and incubated at 37 °C for 6 h. After ZYM A and ZYM B reagents were added to the cell suspension, the mixture was incubated for 10 min. Enzymatic activities are then observed based on color change intensities.

### Determination of vancomycin resistance genes, virulence genes, and biogenic amine genes

The presence of detrimental genes in PMC203 was examined. gDNA was extracted from cultured PMC203 employing a QIAamp DNA Mini Kit (Qiagen, Germany). After checking the extracted DNA concentration, conventional PCR was conducted with gene-specific primers. The amplified PCR products were then subjected to 1.5% agarose gel electrophoresis and observed using a chemidoc XRS system. In this study, the presence of the following genes was investigated: vancomycin resistance genes *vanA* and *vanB*, virulence genes such as *gelE* (gelatinase), *hyl* (hyaluronidase), *ace* (adhesion of collagen), *asa1* (aggregation substance), *efaA* (endocarditis antigen), *cylA* (cytolysin), and *esp* (enterococcal surface protein), and biogenic amines *hdc* (histidine decarboxylase), *odc* (ornithine decarboxylase), and *tdc* (tyrosine decarboxylase) [43–47]. Primer pairs utilized in this study are listed in Table S2.

### Production of biogenic amines and hemolytic activities

The production of biogenic amines in PMC203 was assessed using the method described previously [48]. The probiotic strain was first cultured in MRS broth containing 0.1% of amino acid precursors, such as ornithine, tyrosine, histidine, and lysine (Sigma-Aldrich, Germany). The grown culture was then spread onto MRS agar plates containing the same amino acid precursors. Subsequently, plates were monitored to observe any color changes, with a violet color indicating the presence of biogenic amines. In the hemolytic activity test, the strain was cultured onto Tryptic Soy Agar (Merk, Germany) containing 5% sheep blood defibrinated (MB cells, Korea) and incubated for 48 h at 37 °C. Afterward, complete hemolysis (*α*), partial hemolysis (*β*), or no hemolysis (*γ*) was determined based on clear zones encircling the bacterial culture.

## Results

### Determination of optimal dose based on cytotoxicity

**Fig. 1:**
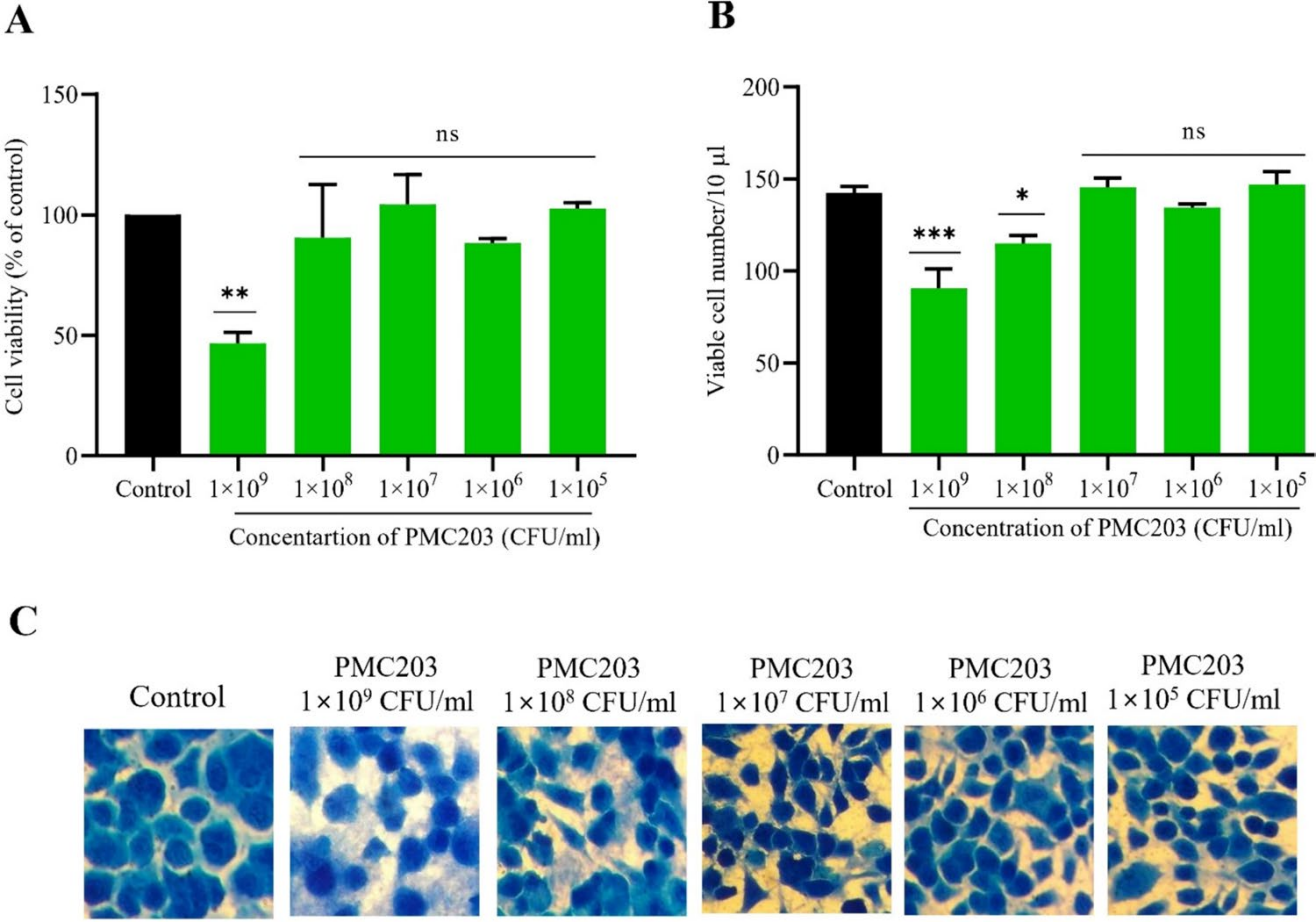
Evaluation of potential cytotoxicity of PMC203 to macrophages**.** Macrophages were incubated with a range of PMC203 concentrations and then cytotoxicity was evaluated employing (**A**) WST (water-soluble tetrazolium salt), and (**B**) trypan blue exclusion assay indicating no cytotoxicity except for higher doses. (**C**) Cell morphology for each concentration treatment condition is presented in light microscope images stained with methylene blue dye. The experiment was performed in triplicate, presenting the results as mean values with corresponding standard deviations. Significance was determined compared to the untreated group using one-way analysis of variance (*p < 0.05; **p < 0.01; ***p < 0.001; ns: non-significant)

After confirming the nonviability of heat killed PMC203, cytotoxicity of the strain to macrophage cells was investigated (Fig. S1, Fig. 1). WST-based cell viability assay showed that cells remained viable after incubating with the probiotic strain at up to 1 × 10^8^ CFU/ml. However, cell viability dropped significantly when the concentration was 1 × 10^9^ CFU/ml (Fig. 1A). The same result pattern was also observed in the trypan blue assay in which cell number decreased remarkably at 1 × 10^8^ and 1 × 10^9^ CFU/ml. However, at other concentrations, cell numbers remain unaltered (Fig. 1B). Furthermore, the methylene blue staining assay showed no noticeable morphological changes or drop in cell viability at concentrations used except for 1 × 10^9^ CFU/ml (Fig. 1C). Based on these experimental findings, we used PMC203 at the maximum concentration of 1 × 10^7^ CFU/ml for forthcoming experiments, which showed no cytotoxicity in any tests.

### Expression levels of LC3‑I, LC3‑II, and p62

**Fig. 2:**
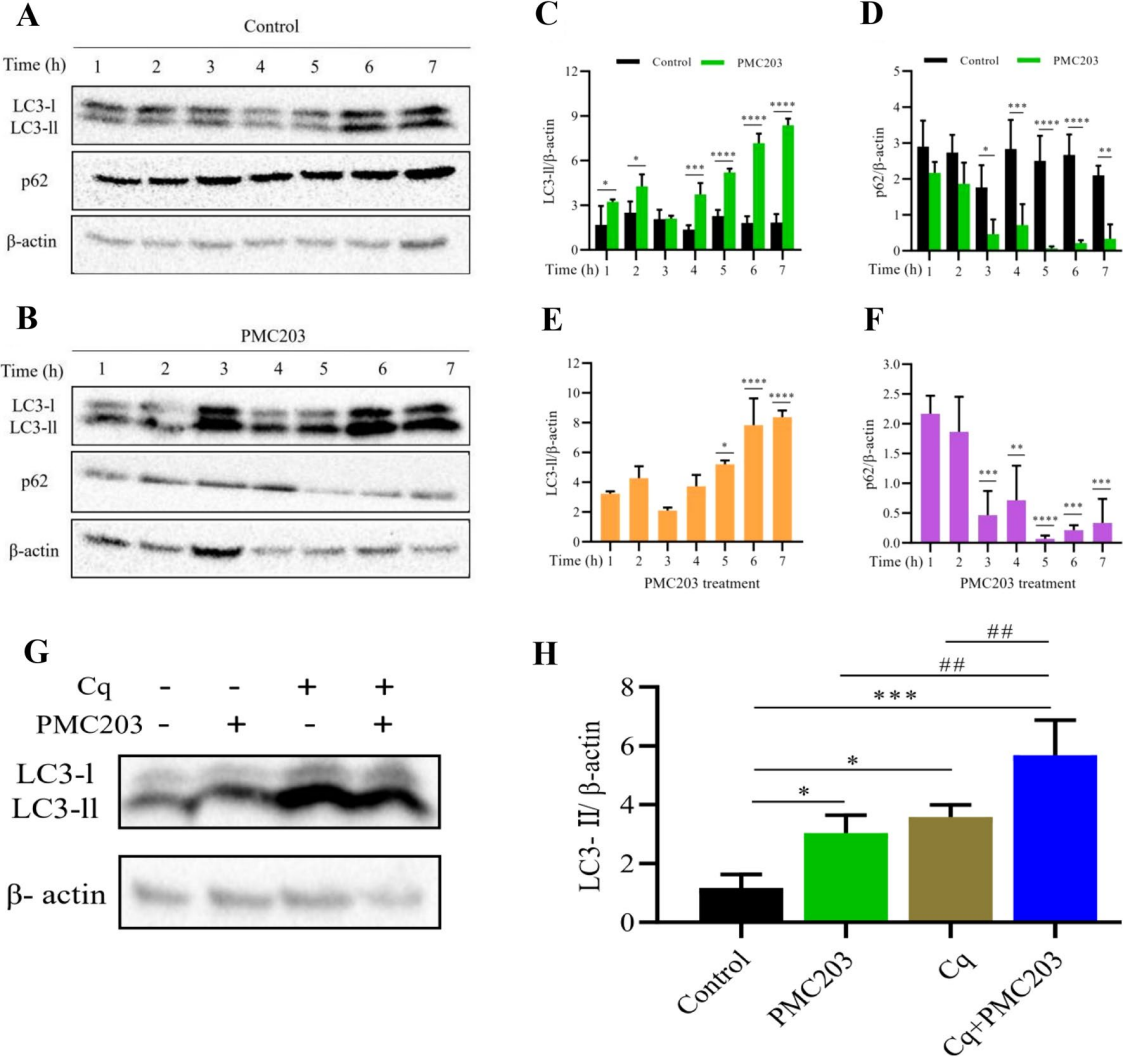
Investigation of PMC203-induced autophagy in macrophage cells. The effect of autophagic flux in response to PMC203 was explored by immunoblotting over time. (**A**, **B**) shows the expression of LC3 and p62, in which visible differences observed in PMC203-treated cells. The obtained protein bands were analyzed by Image J software, showing the increased (**C**) LC3-II to β-actin ratio (**D**) and decreased p62 to β-actin ratio in the treated cells compared to untreated cells. (**E**) LC3-II or (**F**) p62 to β-actin ratio were compared to their corresponding 1 h values, showing a time-dependent autophagy induction by PMC203. (**G**, **H**) Furthermore, autophagy induction was confirmed by using chloroquine (Cq). The experiment was performed in triplicate, presenting the results as mean values with corresponding standard deviations. The statistical significance of the obtained data was determined using with a one-way analysis of variance (*p < 0.05; **p < 0.01; ***p < 0.001; ****p < 0.0001; ^##^*p* < 0.01). *, statistical significance compared with the control; #, denotes statistical significance of PMC203 vs. Cq + PMC203 and Cq vs. Cq + PMC203 treated group

The autophagy induction by PMC203 was explored using macrophage RAW264.7 cells with a western blot technique (Fig. 2, Fig. S2). Cells treated with PMC203 showed a remarkable expression of LC3-II, whereas untreated cells showed no notable expression of LC3-II (Fig. 2A, B). A significant increase in LC3-II to β-actin ratio was noticed within the treated cells compared to untreated cells (Fig. 2C), which started at initial hours (1 and 2 h, *p* < 0.05), and the ratio became more pronounced and more significant as the experimental timeline progressed (at 4 h, *p* < 0.001; 5, 6, and 7 h, *p* < 0.0001). Intracellular LC3-II expression showed a time-dependent increase in cells treated with PMC203 that initiated at 5 h (*p* < 0.05) and consistently maintained elevated levels up to 7 h (*p* < 0.0001) (Fig. 2B, E). Moreover, treated cells showed a decreased expression of p62, which started at 3 h mark (*p* < 0.05) and escalated when time progressed (at 4 h, *p* < 0.001; 5 and 6 h, *p* < 0.0001) in comparison with control cells (Fig. 2D). A significant decrease of this marker in treated cells was also noticed compared to 1 h treatment which initiated at 3 h mark (*p* < 0.001) and intensified as time advanced (at 5, p < 0.0001; 6 and 7 h, *p* < 0.001) in comparison with control cells (Fig. 2F). Furthermore, in the Cq-based assay, the LC3-II to β-actin ratio was significantly increased in the treated group compared to the untreated group. A remarkable statistical difference in terms of LC3-II to β-actin ratio was also observed when Cq + PMC203 treated group was compared to PMC203 (p < 0.01) or Cq (*p* < 0.01) treated group alone (Fig. 2G, H). Altogether, the immunoblot-based findings showed the autophagy-inducing ability of PMC203 as evidenced by elevated expression of LC3-II and reduced levels of p62 over time.

### Immunofluorescence analysis of LC3 and p62

**Fig. 3:**
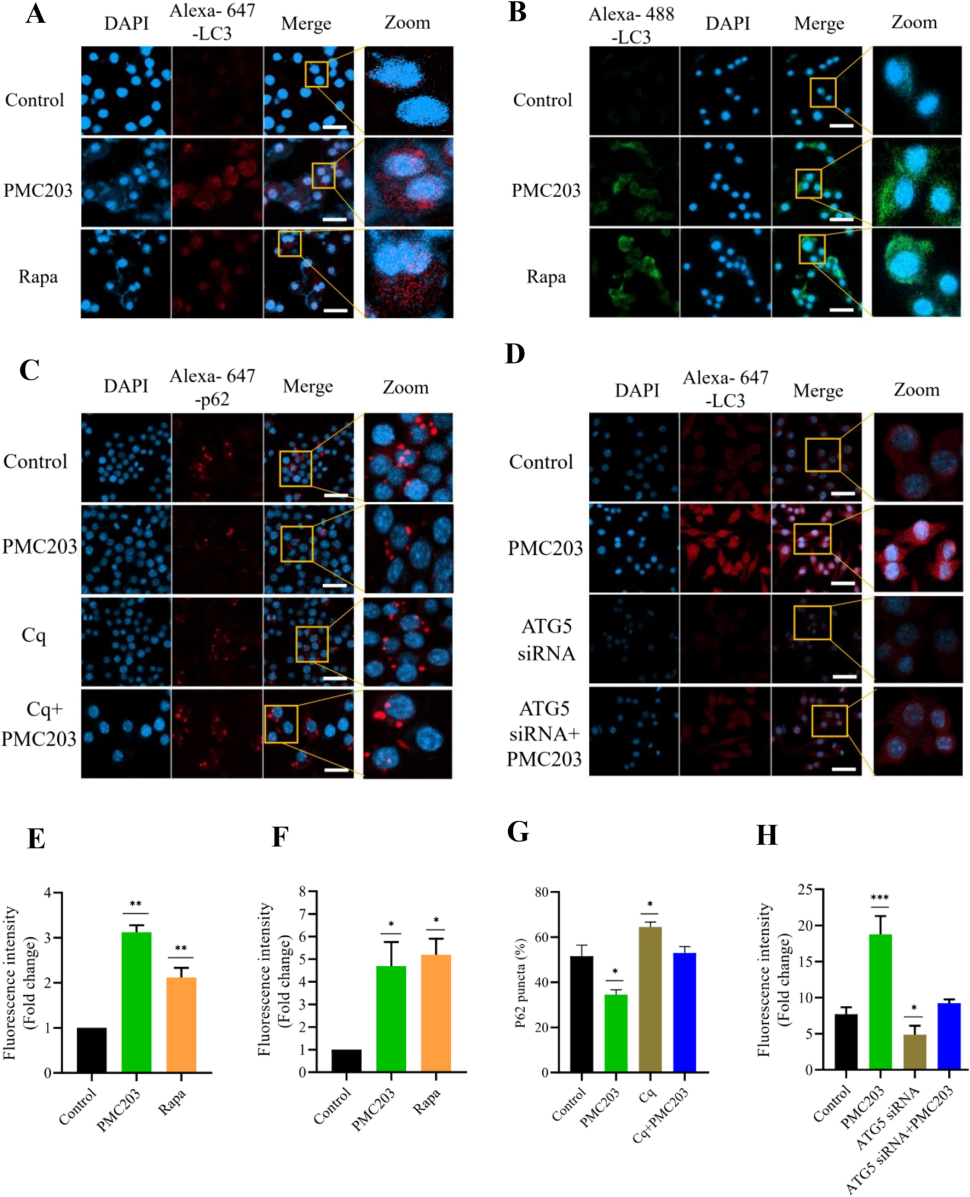
Evaluation of PMC203 mediated autophagic flux based on immunofluorescence. Macrophages were treated with PMC203 or rapamycin (Rapa) and/or chloroquine (Cq) or siRNA. Results show a visual increased of LC3, (**A, D**) red and (**B**) green fluorescence, whereas (**C**) p62 puncta was decreased in PMC203 treated cells compared to untreated cells. In images, the scale bar indicates a length of 10 μm. (**E, F, H**) The fluorescence intensities of the obtained images were analyzed by image J and (**G**) the number of p62 puncta-positive cells was counted from 100 cells per sample. The experiment was performed in triplicate, presenting the results as mean values with corresponding standard deviations. The statistical significance of obtained data was determined compared to the untreated group using a one-way analysis of variance (*p < 0.05; **p < 0.01; ****p < 0.0001)

An immunofluorescence staining was conducted to elucidate PMC203-mediated autophagy further (Fig. 3). Data showed a visible richness and vibrancy of LC3 signal using the alexa-647-LC3 antibody in treated cells compared to untreated cells, where the signal appeared weak and diffused (Fig. 3A). The fold change in fluorescent intensity of obtained images was also significantly higher within treated cells compared to control cells (Fig. 3E). To confirm this finding, we employed the same antibody but coupled it with a distinct fluorescent marker (Alexa-488), and as expected, we observed the same pattern of result (Fig. 3B, F). Furthermore, we explored the degradation of p62. A visible reduction of p62 puncta-positive cells was noticed in PMC203- treated cells. In contrast, the p62 puncta was increased in cells receiving Cq treatment, compared to control cells (Fig. 3C). Calculated p62 puncta-positive cells among cell populations also showed a significant reduction (*p* < 0.05) in the PMC203 treated group compared to the untreated group. Conversely, in the Cq-treated cells, a substantial surge of positive cell populations exhibiting p62 puncta (p < 0.05) was noticed in contrast to control cells (Fig. 3G).

Furthermore, ATG5 siRNA was utilized to assess the impact of PMC203 on autophagy (Fig. 3D, H, Fig. S3). Results showed a decreased expression of LC3 in the siRNA-treated cells whereas an increased vibrancy of the marker was noticed in the PMC203 treated cells. The fold change in fluorescent intensity of obtained images was also significantly higher within PMC203- treated cells than untreated cells. Altogether, these results further confirm the autophagy stimulating-capability of PMC203 characterized by increased LC3 signal and decreased p62 puncta.

### Formation of autophagic vacuole

**Fig. 4:**
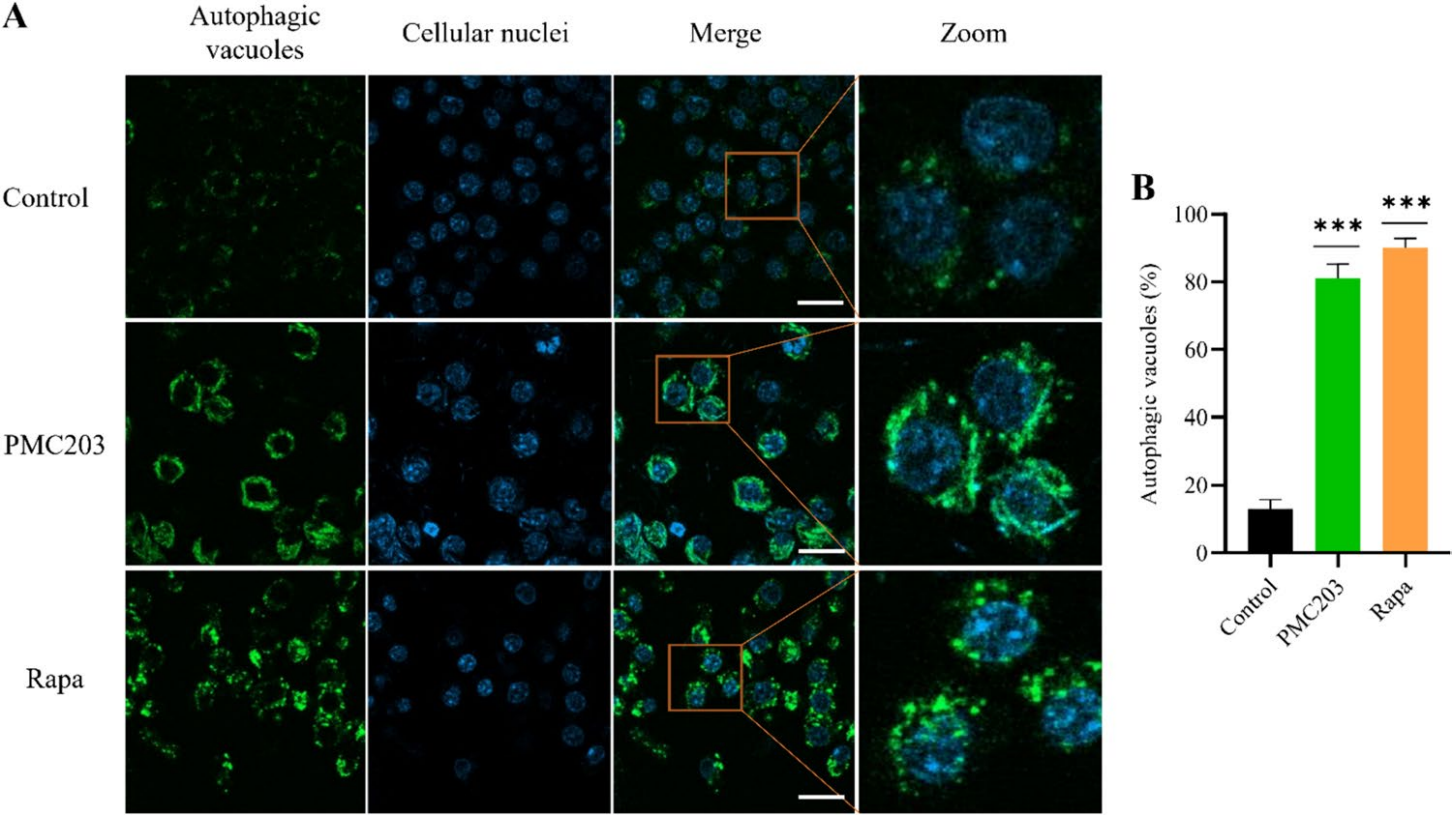
Determination of PMC203-induced autophagic vacuole formation. (**A**) The effects of PMC203 on the formation of LC3 and vesicle colocalization were measured, indicating an elevated visual expression of green fluorescence compared to an untreated group. The scale bar in these images indicates a length of 10 μm. (**B**) The number of positive cells with > 3 green puncta was calculated, with data obtained from 100 cells per sample. The experiment was performed in triplicate. Results are presented as mean values with corresponding standard deviations. The statistical significance of obtained data compared to an untreated group was determined using a one-way analysis of variance (****p < 0.0001)

The autophagic vacuole formation was investigated upon treating cells with PMC203 (Fig. 4). Results showed a heightened intensity with vivid autophagic vacuoles co-localized with LC3 in cells subjected to PMC203/Rapa treatment (Fig. 4A). On the other hand, diffuse and faint autophagic vacuoles were manifested in untreated cells. Additionally, the autophagic vacuole formation rate was significantly increased in PMC203-treated cells in comparison with control cells (*p* < 0.001) (Fig. 4B). These findings demonstrate the formation of autophagic vacuoles in response to PMC203 treatment leading to the progression of the autophagic process.

### Lysosomal biogenesis formation

**Fig. 5:**
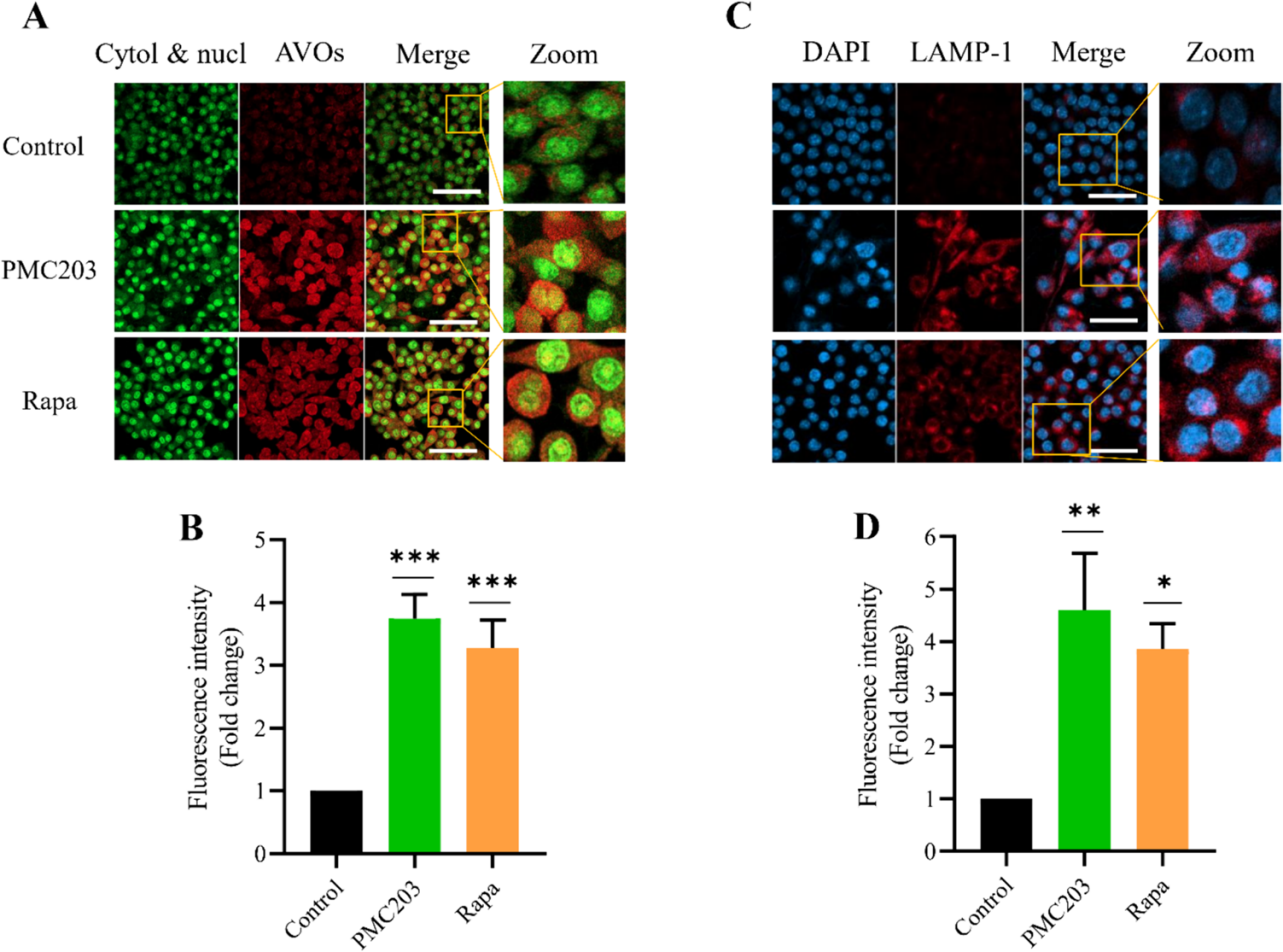
Assessment of PMC203-induced lysosomal biogenesis formation. The generation of lysosomal biogenesis in response to PMC203 treatment was investigated, showing a visual increase of (**A**) AVOs and (**C**) LAMP1 in the treated cells compared to the control. In these images, the scale bar represents 10 µm. (**B**, **D**) Image J analysis of fluorescence intensities of the obtained images. The experiment was performed in triplicate. Results are presented as mean values with corresponding standard deviations. The statistical significance of the obtained data compared to an untreated group was determined using a one-way analysis of variance (**, *p* < 0.01; ****, *p* < 0.0001)

Moreover, the acquisition of LAMP1, a lysosomal biogenesis marker, and the formation of acidic vesicular organelles were examined after PMC203 treatment (Fig. 5). Cells treated with PMC203 or Rapa showed an intensified red fluorescence signal exhibiting acidic molecule and LAMP1, respectively. However, this signal was feeble and weak in untreated cells (Fig. 5A, C). Additionally, a quantitative analysis of fluorescence intensity demonstrated significant increases in fold change for AVOs (*p* < 0.001) and LAMP1 (*p* < 0.01) in treated cells compared to untreated cells (Fig. 5B, D). In conclusion, PMC203 treatment results in the increased formation of lysosomal biogenesis.

### Reduction of *M. tuberculosis* burden in macrophages by PMC203-induced autophagy

**Fig. 6:**
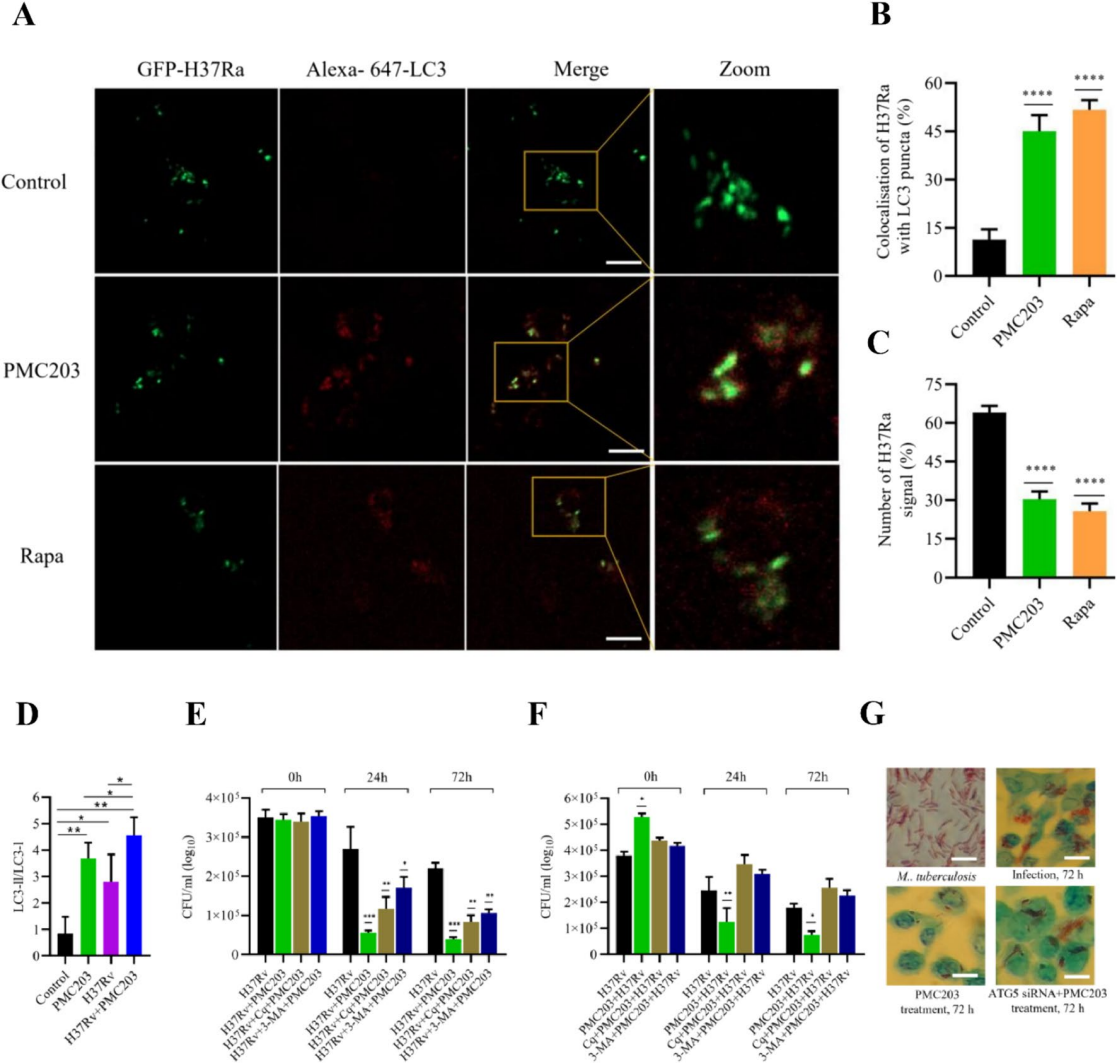
Evaluation of *M. tuberculosis* reduction through induction of autophagy by PMC203. **(A)** Macrophages were infected, treated with PMC203, and colocalization of GFP-H37Ra with LC3 puncta was observed, indicating increased colocalization in the treated group. (**B**) Meanwhile, the number of positive cells with > 1 colocalization was calculated using image J. (**C**) At the same time, the number of positive cells containing > 6 GFP-H37Ra signal was calculated, in which H37Ra signal was reduced in the treated group compared to the untreated group. In these images, the scale bar represents 10 µm. (**B**, **D**) Image J analysis of fluorescence intensities of the obtained images. (**D**) The conversion of LC3-I to LC3-II was examined using an immunoblot assay, showing a significant statistical difference between the groups. To observe the (**E**) bactericidal and (**F**) phagocytosis effect, CFU was determined in a time-dependent manner using *M. tuberculosis-*specific Middlebrook H710 agar media, showing the reduction of H37Rv in the PMC203-treated groups, whereas the reduction was disrupted in the Chloroquine (Cq)/3-methyladenine (3-MA) treated cells. (**G**) Additionally, the impact of PMC203 induced autophagy on *M. tuberculosis* survival was evaluated with siRNA using the acid-fact bacilli staining method. The images of acid-fast bacilli staining include a scale bar representing 10 µm. (**B**, **D**) Image J analysis of fluorescence intensities of the obtained images. The experiment was performed in triplicate. Results are presented as mean values with corresponding standard deviations. The statistical significance of the obtained data was determined compared to the untreated group using one-way analysis of variance (*p < 0.05; **p < 0.01; ****p < 0.0001; ^#^*p* < 0.05)

The effect of PMC203-induced autophagy on the clearance of *M. tuberculosis* was explored (Fig. 6). Results showed visible GFP-H37Ra-LC3 colocalization structures in treated cells, while faint colocalization was noticed in the control group (Fig. 6A). The number of positive cells with > 1 colocalization was also calculated, revealing an elevated colocalization rate in treated groups compared to the untreated group (*p* < 0.0001) (Fig. 6B). Moreover, the number of positive cells containing > 6 GFP-H37Ra signal was determined, demonstrating a significant reduction of *M. tuberculosis* signal (*p* < 0.0001) in treated groups (Fig. 6C). In the immunoblotting assay, a significant increase in the conversion from LC3-I to LC3-II was noticed within the treated groups compared to the control. A significant statistical difference was also observed when the H37Rv + PMC203 treated group was compared to the group treated with H37Rv alone (*p* < 0.05) (Fig. 6D). Similarly, there was a significant difference noticed between PMC203 and the combination of PMC203 and H37Rv (*p* < 0.05).

Results of the bactericidal assay revealed that H37Rv counts were similar upon phagocytosis at 0 h of treatment across all groups and then significantly reduced at 24 h and 72 h of post-treatment with heat-killed nonviable PMC203 (*p* < 0.001) (Fig. 6E, Fig. S4). Interestingly, when infected cells were treated with Cq or 3-MA followed by treatment with PMC203, the antibacterial effectiveness dropped considerably, although there was still a significant bacterial reduction, albeit not to the level noticed with PMC203 alone. Phagocytosis and subsequent antibacterial activities were also examined in which cells were initially treated with PMC203 or Cq + PMC203/3-MA + PMC203 and then subjected to H37Rv treatment. The measurement of phagocytosed bacteria showed an increased uptake of *M. tuberculosis* in the PMC203-treated cells compared to the control. However, following 24 h (*p* < 0.01) and 72 h (*p* < 0.05) of post-treatment, intracellular bacteria were reduced significantly in the probiotic-treated cells in comparison to untreated cells. On the other hand, in the group treated with Cq or 3-MA following PMC203 treatment, bactericidal activity was decreased, and an elevated CFU was evident (Fig. 6F, Fig. S5). Moreover, siRNA was also utilized to explore the effect of PMC203-mediated autophagy in *M. tuberculosis* killing. The AFB staining showed a visible growth of *M. tuberculosis* in the siRNA- treated cells, whereas a remarkable decrease was observed in the PMC203-treated cells (Fig. 6G).

Furthermore, to confirm the PMC203-mediated autophagy in reducing *M. tuberculosis* load, a new anti-*M. tuberculosis* model was explored using a different cell type, J774A.1 (Fig. S7). Similar results were observed with J777.A1 cells as with RAW264.7. A detailed procedure for this new model was described in the supplementary material. However, altogether, these results demonstrate that PMC203 provokes autophagy and subsequently plays a role in reducing *M. tuberculosis* load in macrophage cells.

### Autophagy-related gene expression

**Fig. 7:**
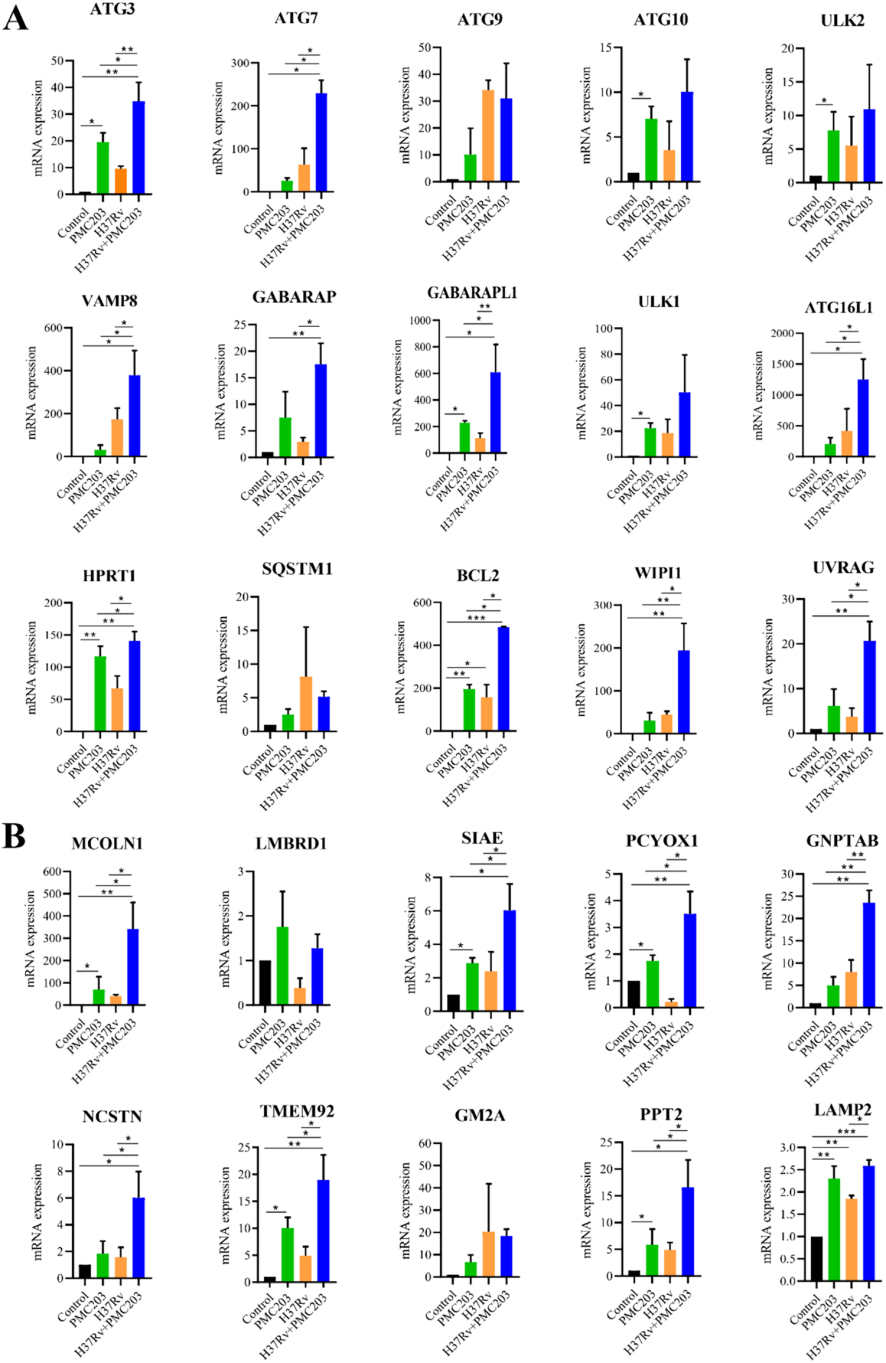
Measurement of PMC203-induced autophagic gene expression. RT-PCR assay revealed a significant upregulation of most of the (**A**) autophagy induction and (**B**) lysosomal biogenesis genes in cells treated with H37Rv + PMC203, while H37Rv treatment alone failed to reach significant levels, both in comparison with the control. The experiment was performed in triplicate. Results are presented as mean values with corresponding standard deviations. The statistical significance of the obtained data was determined compared to the untreated group using a one-way analysis of variance (*p < 0.05; **p < 0.01)

The mRNA expression levels of autophagy induction and lysosomal biogenesis genes were examined (Fig. 7). Results showed that expression levels of autophagy induction genes were markedly increased after treatment with PMC203 alone or H37Rv + PMC203 but not after H37Rv treatment alone, compared to the control group (Fig. 7A). It was found that mRNA levels of genes were remarkably elevated after treatment with PMC203 alone or with PMC203 in conjunction with H37Rv. Particularly, genes, including ATG3, ATG7, VAMP8, GABARAP, GABARAPL1, ATG16L1, HPRT1, BCL2, WIPI1, and UVRAG were significantly increased after treatment with H37Rv + PMC203. Similarly, except GABARAP, these genes were significantly upregulated while comparing PMC203 alone with H37Rv + PMC203 group. A significant difference was also noticed for these genes between the H37Rv alone and the H37Rv + PMC203 group. Although expression levels of other genes were also increased, their increases were not statistically significant. No significant expression of genes was noticed when cells were exposed to H37Rv alone compared to the control.

We also determined mRNA expression levels of lysosomal biogenesis genes in response to PMC203 treatment (Fig. 7B). Cells exposed to PMC203 or H37Rv + PMC203 exhibited significant expression of genes. Notably, genes, including MCOLN1, SIAE, PCYOX1, GNPTAB, NCSTN, TMEM92, PPT2, and LAMP2, were significantly increased compared to control cells. Besides, except LAMP2, these genes were significantly elevated while comparing PMC203 alone and H37Rv + PMC203. A significant difference was also observed for these genes between the infected group and treated group. On the other hand, there was no remarkable expression of genes except for LAMP2 when cells were exposed to H37Rv alone. These findings show that PMC203 can significantly upregulate the autophagy gene expression in macrophages when treated alone or co-treated with *M. tuberculosis*.

### Reactive oxygen species (ROS) generation

The impact of PMC203 on the generation of ROS within macrophages was explored (Fig. S6). Data showed a significant increase in ROS level when cells were treated with menadione (used as a positive control) or H37Rv in comparison with the control group (*p* < 0.001). Interestingly, upon treating infected cells with the probiotic strain PMC203, a significant reduction (*p* < 0.01) in ROS level was observed compared to infected cells without probiotic treatment, indicating that the PMC203 strain could maintain the ROS level within macrophage cells.

### Enzyme production, detrimental genes, biogenic amines, and hemolysis activities

Table S3 shows that PMC203 can produce acid phosphatase, β-galactosidase, α- glucosidase, and 6-Br-2-naphtyl-β-D-glucopyranoside enzymes. On the other hand, the following enzymes, including esterase lipase (c8), esterase (c4), valine acrylamidase, alkaline phosphatse, and leucine arylamidase, were produced moderately. It was also evident that PMC203 did not produce α-mannosidase, α-fucosidase, N-acetyl- β-glucosaminidase, or β- glucuronidase. The presence of vancomycin resistance, virulence, and biogenic amine genes in the PMC203 strain was evaluated (Table S4). We did not observe the presence of vancomycin resistance genes (vanA or vanB), virulence genes (cylA, gelE, efaA, hyl, asa1, or ace), or biogenic amines-producing genes (hdcA, tdcA, or odcA) in the PMC203 strain. The production of biogenic amines and hemolytic activities were checked using an agar medium. The strain did not generate biogenic amines, including histamine, ornithine, lysine, or tyrosine. Additionally, it displayed no α-hemolytic (complete hemolysis) or β-hemolytic (partial hemolysis) activities, instead showing γ activities on blood agar medium. Altogether, these results show a specific enzyme production pattern and hemolytic activities of the PMC203 strain and also reveal the absence of detrimental genes.

## Discussion

Despite extensive attempts to control and eradicate TB, it remains a prominent global public health concern. The current drug regime used for TB treatment has many side effects [49], as well as the emergence of drug-resistant strains further complicates TB control efforts [50]. This pressing concern demands the development of a novel anti-TB drug, which led us to explore the potential of a probiotic strain, *Lactocaseibacillus rhamnosus* PMC203, to reduce *M. tuberculosis* burden through stimulation of autophagy, an essential component of the body’s natural defense system.

Recently, probiotics have been recognized as a promising therapeutic option in medicine against various human diseases beyond their traditional roles as food supplements [51]. Regarding this, different mechanisms have been proposed behind their positive effects on hosts to treat diseases, of which autophagy has been recognized as a major one. It has been found that probiotic strains can stimulate autophagy, thus protecting the host against a variety of pathogenic infections [21]. Contemporaneously, the role of autophagy in the context of TB has gained remarkable attention as it can function as a defense mechanism inhibiting the survival of this deadly pathogen [33]. Hence, the present study, we focused on probiotic-mediated autophagy and its subsequent role in reducing the *M. tuberculosis* burden.

Our current study used heat-killed, nonviable probiotic strain PMC203 based on studies showing the ability of heat-killed bacteria to stimulate autophagy. For instance, Wu et al., 2017 demonstrated that heat-killed Probiotic *Bacillus amyloliquefaciens* SC06 can stimulate autophagy and protect macrophages against *Escherichia coli* [52]. Likewise, another study showed that heat- killed *Lactobacillus plantarum* markedly triggered autophagy in response to *Salmonella* intracellular infection [53]. Therefore, the potentiality of heat killed probiotic bacteria in triggering autophagy led us to utilize heat-killed PMC203 in our study.

Results obtained from this study demonstrated stimulation of autophagy by PMC203 within macrophages, revealing a time-dependent progression of autophagic markers. All results were achieved with a non-cytotoxic dose range. The elevation of microtubule-associated protein 1 light chain 3 (MAP1LC3-II/LC3-II), a dependable autophagy marker, following PMC203 treatment indicated autophagic progression, in line with earlier findings [54]. The reduction of p62 (also known as SQSTM1/sequestosome 1) expression in PMC203-treated cells further strengthened the autophagy-inducing effects of the strain, as phagosome-lysosome fusion could enhance p62 degradation [55].

Moreover, a significant upregulation of the LC3-II to β-actin ratio in cells treated with PMC203, particularly in the presence of Cq, a lysosomal biogenesis inhibitor, demonstrated the autophagy stimulation ability of the probiotic strain. This findings aligns with a previous study that reported the accumulation of LC3-II in the presence of a lysosomal inhibitors indicating an enhanced autophagic flux [56]. Besides, a significant accumulation of LC3-II in the Cq treated group compared to control group confirms the effectiveness of Cq as an autophagy inhibitor corroborating previous research [57]. Cq can block the late steps of autophagic pathway, such as, autophagosome fusion with lysosomes and/or lysosomal degradation leading to the accumulation of LC3-II [57]. This accumulation occurs because LC3-II tightly bound with autophagosomal membranes membrane [58].

Immunofluorescence-based confocal images with LC3 antibodies revealed that PMC203 treatment led to a discernible increase of LC3 fluorescent intensity in treated cells, which aligned with established indicators of autophagy initiation and progression [59]. The results of this study also corroborate the findings of Rapa, a widely used autophagy inducer that can increase the LC3 fluorescent signal in macrophage cells [60]. It was found that p62 degradation was decreased in cells subjected to treatment with Cq. This agent could block the degradation of p62 and result in its accumulation [61], further underscoring autophagy stimulation by PMC203. The results of ATG5 siRNA further demonstrate the autophagy-inducing ability of our strain. ATG5 siRNA can particularly block the transcriptional activities of ATG5, which is the essential protein involved in the autophagy process and regulates the autophagosome formation, in inhibiting the autophagy machinery [62, 63]. The findings of our study are consistent with previous research, which demonstrated a decreased expression of LC3 in cells subjected to ATG5 siRNA treatment [62, 64]. Additionally, the formation of autophagic vacuoles was confirmed by increased detection of a green detection fluorescent probe [65].

Acidic vesicular organelles (AVOs), as indicators of active autophagy [66], were increased in response to PMC203 treatment. Moreover, LAMP1, belonging to a group of integral proteins of lysosomal membranes crucial for lysosomal biogenesis [67], was found to be elevated upon PMC203 treatment. Taken together, the combined increase of LAMP1 and AVOs upon PMC203 treatment could infer that the production of phagolysosomes known to be acidic organelles formed as a result of fusion between phagosomes and lysosomes is responsible for the degradation of internalized material.

Findings from our study underscore the substantial impact of PMC203-induced autophagy on the clearance of *M. tuberculosis* in macrophage cells. Immunofluorescence-based confocal images revealed the capability of the probiotic strain to form colocalization between GFP-H37Ra and LC3, indicative of autophagy stimulation by PMC203, which is in line with previous research findings [29]. A significant reduction of GFP-H37Ra signal in treated cells further signified the impact of PMC203-induced autophagy to target and eliminate intracellular *M. tuberculosis*, aligning with an earlier study reporting that physiological or pharmacological stimulation of autophagy could suppress survival of this lethal pathogen [33]. A separate study showed that poly(I:C)-mediated autophagy could contribute to the clearance of mycobacteria within macrophages [68], further supporting our current findings. Furthermore, an increase of LC3-I to LC3-II conversion in the PMC203 + H37Rv group compared with the H37Rv alone indicates the synergic effect of PMC203 with H37Rv and also reflects the autophagy concerning the probiotic strain. This finding aligns with a previous study that showed increased autophagy when cells were treated with probiotics and pathogens compared to pathogen treatment alone [53]. Besides, increased autophagy while comparing PMC203 alone with PMC203 and H37Rv further highlights the ability of the probiotic strain to trigger autophagy in conjunction with H37Rv.

A colony-forming unit assay further confirmed the reduction of *M. tuberculosis* load within infected macrophages, consistent with previous findings [52]. However, the introduction of Cq diminished bactericidal effectiveness. Cq is an FDA-approved chemical mainly used to inhibit autophagy by impairing autophagosome fusion with lysosomes [69]. One study has shown that Cq can induce severe disorganization of the endo-lysosomal system, further contributing to fusion impairment, whereby this fusion formation is vital for pathogen elimination [57]. An elevated number of bacteria in cells initially treated with Cq and PMC203 followed by H37Rv treatment indicated phagocytosis activities of the probiotic strain, which aligns with a previous study [29]. Furthermore, we also utilized another most widely used pharmacological autophagy inhibitor, 3-MA, to confirm the PMC203-induced anti-*M. tuberculosis* activities. 3-MA is a class III phosphatidylinositol 3-kinase (PtdIns3K) inhibitor that acts on a different autophagic progression stages compared to Cq, making its function distinct [70]. Specifically, 3-MA blocks the autophagic activation through inhibiting the formation of PtdIns3K, crucial autophagy initiation [71], while Cq blocks the later stage of autophagic progression, particularly phagolysosomal formation [69]. However, as expected, we observed the same pattern of results as we noticed in the case of Cq. In addition, to ensure the effect of our strain ATG5 siRNA was utilized. An increase in *M. tuberculosis* was noticed in the siRNA-treated group, whereas bacterial load was decreased in the PMC203-treated groups. These results are in line up with a previous study that showed the impairment of autophagy with ATG5 siRNA favored the survival of H37Ra in RAW264.7 cells [72]. Moreover, we investigated the impact of PMC203 on reducing the *M. tuberculosis* burden employing a different cell line, J77A4.1, in addition to RAW264.7. J77A4.1 is also a mouse macrophage cell commonly used in immunomodulation research [73] and become a valuable tool for studying TB pathogenesis [74, 75]. Expectedly, we observed a similar pattern of results with J774A.1 cells, as we noticed with RAW264.7. Thus, our findings ensured that PMC203-mediated autophagy could contribute eliminating of intracellular *M. tuberculosis* in macrophage cells. Furthermore, we can conclude from these results that PMC203 strain affects macrophages results in increased antituberculosis effect. These observations are consistent with some previous studies demonstrating that specific probiotic bacteria can enhance the antimycobacterial activity of macrophages [6, 76].

We also investigated the expression of autophagy induction genes and lysosomal biogenesis genes upon H37Rv and/or PMC203 treatment. Data revealed a significant increase of most genes in the PMC203 alone treated group, consistent with previous studies [29, 77], while revealing no significant difference in the H37Rv alone group compared to the control group. A significant difference was also noticed between PMC203 alone and H37Rv + PMC203 group. Besides, the transcription levels of autophagy-related genes were significantly altered between H37Rv alone and H37Rv + PMC203 group. These results indicate a unique ability of PMC203 to influence the autophagy-related transcriptional activities, as evidenced by the elevated levels of gene expression. During the autophagic process, the formation of autophagosomes, extension of isolated membranes, and/or completion of enclosure are facilitated due to the expression of autophagy induction genes. In contrast, lysosomal biogenesis formation is promoted through the participation of lysosomal biogenesis genes [78].

Excessive ROS production, hemolytic activity, synthesis of biogenic amines, enzymatic activities, and virulence genes are potential factors that can cause cellular damage and induce autophagy as a standard cellular defense mechanism [79–84]. Hence, we investigated the toxicity and safety of the probiotic strain in terms of cellular damage with drug development considerations in the future. Data showed that PMC203 maintained ROS production and exhibited no hemolytic activity. It did not possess antibiotic resistance, biogenic amine, or virulence genes. Additionally, this probiotic strain did not contain the β-glucuronidase enzyme involved in cancer development [85]. These findings indicate that autophagy induction by PMC203 is related to its intended effects. They also emphasize the safety profiles of this probiotic strain for future drug development.

To summarize, the findings of this study showed the impact of PMC203 on autophagy induction and, subsequently, its capability in combating *M. tuberculosis*. However, further in-depth research studies, including in vivo animal experiments, must confirm its efficacy and safety. Despite some shortcomings, we hope these findings will be valuable to probiotics, autophagy, and tuberculosis, as they help develop microbiome-based innovative therapeutic approaches against tuberculosis.

## Conclusions

Our findings indicate that PMC203 can stimulate autophagy, evidenced by reliable autophagy markers with heightened expression of autophagy-related genes, thus contributing to the subsequent reduction of *M. tuberculosis* in macrophages. Strikingly, to the best of our knowledge, this is the first study demonstrating the role of probiotics in promoting autophagy for *M. tuberculosis* clearance within macrophages. Findings obtained from this study emphasize the potential of using the PMC203 strain as a valuable therapeutic intervention against intracellular pathogens. However, the practical functioning of its protective mechanisms within a living organism requires further comprehensive exploration.

## Supplementary Information

Below is the link to the electronic supplementary material.

Supplementary file1 (DOCX 6809 KB)

## Data Availability

Data will be made available upon reasonable request.
