# Supplementary material for: Exploring the potential of Lactocaseibacillus rhamnosus PMC203 in inducing autophagy to reduce the burden of Mycobacterium tuberculosis
Source: Med Microbiol Immunol. 2024 Jul 9;213(1):14. doi: 10.1007/s00430-024-00794-z (PMC11231020; doi:10.1007/s00430-024-00794-z)
Supplement: Supplementary file 1 — Supplementary file1 (DOCX 6809 KB) [file 430_2024_794_MOESM1_ESM.docx]

Supplementary Information

**Table S1.** List of primers for autophagy induction genes and lysosomal biogenesis genes

| **Category** | **Gene name** | **Forward (5`→3`)** | **Reverse (5`→3`)** |
| --- | --- | --- | --- |
| Autophagy induction genes | ATG3 | GGCGGATGGGTAGATACATAT | GCTTCTCCTTCATCTTCATCTTC |
|  | ATG7 | TGAGTTGACCCAGAAGAAGCT | CCCAGCAGAGTCACCATTGT |
|  | ATG9 | TTTGCTCAGATGGATGTTCG | TCTTTCCATCCTCAGCTTGC |
|  | ATG10 | GATAGGTGATAGTTGGGAATGG | TGGGTAGATGCTCCTAGATG |
|  | ATG16L1 | GACAGGCGTTCGAGGAGATC | TCAGCCTGTAGTTTCTGGGC |
|  | BCL2 | TGTGGATGACTGAGTACCTG | GCCAGGAGAAATCAAACAGAG |
|  | VAMP8 | CATCTCCGCAACAAGACAGA | CTTCACGTTCTTCCACCAGAA |
|  | GABARAP | GAAGAGCATCCGTTCGAG | TGAGATCAGAAGGCACCAG |
|  | ULK1 | ACAAGAAGAACCTCGCCAAG | TTCCTTCAGGATTTTGATTTCC |
|  | ULK2 | CTGACTTGTGGAGCATAGG | AATAAGGTGATGTTTCTCTGGG |
|  | SQSTM1 | GGAGCAGATGAGGAAGATCG | TGGGTCCAGTCATCATCTCC |
|  | UVRAG | TCAACATGGACTAGGGACTC | ACTGGAGGTGTGATGTCTG |
|  | GABARAPL1 | GAGTGTCTATGGGAAATGAGTG | TGTCTTCACCTTCTGTCTCC |
|  | HPRT1 | TGACACTGGCAAAACAATGCA | GGTCCTTTTCACCAGCAAGCT |
|  | WIPI1 | TGTTGAAGACCCTCCTGGAT | TTCCAGGATAGGCCAGGTAA |
| Lysosomal biogenesis genes | MCOLN1 | AGGGGCTCTGGGCTACC | GCCCGCCGCTGTCACTG |
|  | GM2A | TGATCAGAAGCCTGACTCTG | AGGTACAGCTGCCAATGTAG |
|  | GNPTAB | ATGGAGCCGAGATCAATACC | CTGTAGTTCCTTCAGTAGTTC |
|  | LAMP2 | TGTGCGGTCTTATGCATTGG | TTCTGATCATCCCCACAAATG |
|  | LMBRD1 | ACCCTTTCTGTGCCAAAGAG | AAGGCCCAGTTACCAAAATAG |
|  | NCSTN | CAACGCCACTCATCAGATTG | AAATGCTTGCTCTCCAGCAG |
|  | PCYOX1 | GACGTGTTAGACAAGTTCATG | GGAGCAATCATTTCATTGAGG |
|  | PPT2 | GGTCTAACCTCTATCGGATC | GTGGCATTGGGATGGTCTC |
|  | SIAE | TGCGCCAAGGTCAGGAAAC | CCAAAGTCTGTTGTGCCATC |
|  | TMEM92 | AAGGATTCAAATGCTGTGGTG | GTTGCGACAGAAGCACTTAG |

**Table S2.** List of primers for vancomycin resistance genes, virulence genes, and biogenic amines genes

| Vancomycin resistance | van-AF | TCTGCAATAGAGATAGCCGC | GGAGTAGCTATCCCAGCATT |
| --- | --- | --- | --- |
|  | van-BF | GCTCCGCAGCCTGCATGGACA | ACGATGCCGCCATCCTCCTGC |
| Virulence genes | gel11-F | TATGACAATGCTTTTTGGGAT | AGATGCACCCGAAATAATATA |
|  | hyl n1-F | ACAGAAGAGCTGCAGGAAATG | GACTGACGTCCAAGTTTCCAA |
|  | asa11-F | GCACGCTATTACGAACTATGA | TAAGAAAGAACATCACCACGA |
|  | esp14-F | AGATTTCATCTTTGATTCTTGG | AATTGATTCTTTAGCATCTGG |
|  | cyt Ib | ACTCGGGGATTGATAGGC | GCTGCTAAAGCTGCGCTT |
|  | efa-AF | GCCAATTGGGACAGACCCTC | CGCCTTCTGTTCCTTCTTTGGC |
|  | ace F | GAATTGAGCAAAAGTTCAATCG | GTCTGTCTTTTCACTTGTTTC |
| Biogenic amines | JV16HC | AGATGGTATTGTTTCTTATG | AGACCATACACCATAACCTT |
|  | P2-F | GAYATNATNGGNATNGGNYTNGAYCARG | CCRTARTCNGGNATAGCRAARTCNGTRTG |
|  | Orn3-F | GTNTTYAAYGCNGAYAARACNTAYTTYGT | ATNGARTTNAGTTCRCAYTTYTCNGG |

**Table S3:** Enzymatic activities of PMC203 strain

| No. | Enzyme | Substrate | Scores |
| --- | --- | --- | --- |
| 1 | Control |  | 0.0 |
| 2 | Alkaline phosphatase | 2-naphthyl phosphate | 3.0 |
| 3 | Esterase (C4) | 2-naphthyl butyrate | 3.0 |
| 4 | Esterase lipase (C8) | 2-naphthyl caprylate | 3.0 |
| 5 | Lipase (C14) | 2-naphthyl myristate | 1.5 |
| 6 | Leucine arylamidase | L-leucyl-2-naphthylamide | 3.0 |
| 7 | Valine arylamidase | L-valyl-2-naphthylamide | 3.0 |
| 8 | Cystinearylamidase | L-cystyl-2-naphthylamide | 2.0 |
| 9 | Trypsin | N-benzoyl-DL-arginine-2-naphthylamide | 0.5 |
| 10 | α-chymotrypsin | N-glutaryl-phenylalanine-2-naphthylamide | 1.5 |
| 11 | Acid phosphatase | 2-naphthyl phosphate | 5.0 |
| 12 | Naphthol-AS-BI-phosphohydrolase | Naphthol-AS-BI-phosphate | 2.0 |
| 13 | α -galactosidase | 6-Br-2-naphthyl-a D-galactopyranoside | 1.0 |
| 14 | β-galactosidase | 2-naphthyl-b D-galactopyranoside | 4.0 |
| 15 | β -glucuronidase | b-glucuronidase | 0.0 |
| 16 | α -glucosidase | 2-naphthyl-a D-glucopyranoside | 4.0 |
| 17 | 6-Br-2-naphthyl-b D-glucopyranoside | 6-Br-2-naphthyl-b D-glucopyranoside | 5.0 |
| 18 | N-acetyl-b-glucosidaminidase | 1-naphthyl-N-acetyl-b D-glucosamide | 0.0 |
| 19 | α -mannosidase | 6-Br-2-naphthyl-a D-mannopyranoside | 0.0 |
| 20 | α -fucosidase | 2-naphthyl-a L-fucopyranoside | 0.0 |

Numerical values represent the color change intensity; 0, no activity; 5, maximum activity

**Table S4.** Determination of the presence of detrimental genes, biogenic amines, and hemolytic activities in PMC203

| **Category** | **Target genes** | **Result** |
| --- | --- | --- |
| Vancomycin resistance gene | *van*A (Vancomycin resistance) | - |
|  | *van*B (Vancomycin resistance) | - |
| Virulence genes | *gel*E (gelatinase) | - |
|  | *hyl (*Hyaluronidase) | - |
|  | *asa*1 (Aggregation substance) | - |
|  | *ace* (Adhesin of collagen protein) | - |
|  | *efa*A (Endocarditis antigen) | - |
|  | *cyl*A (Cytolysin (*cylA*) | - |
|  | *esp* (Enterococcal surface | - |
| Biogenic amine genes | *hdcA* (Histidine decarboxylase) | - |
|  | *tdcA (*Tyrosine decarboxylase) | - |
|  | *odcA* (Ornithine decarboxylase) | - |
| **Category** | **Target product** | **Result** |
| Biogenic amines | histidine | - |
|  | ornithine | - |
|  | lysine | - |
|  | tyrosine | - |
| Hemolytic activity | (*α*, *β*, *γ*) | *γ* |

“-” indicates the absence of it


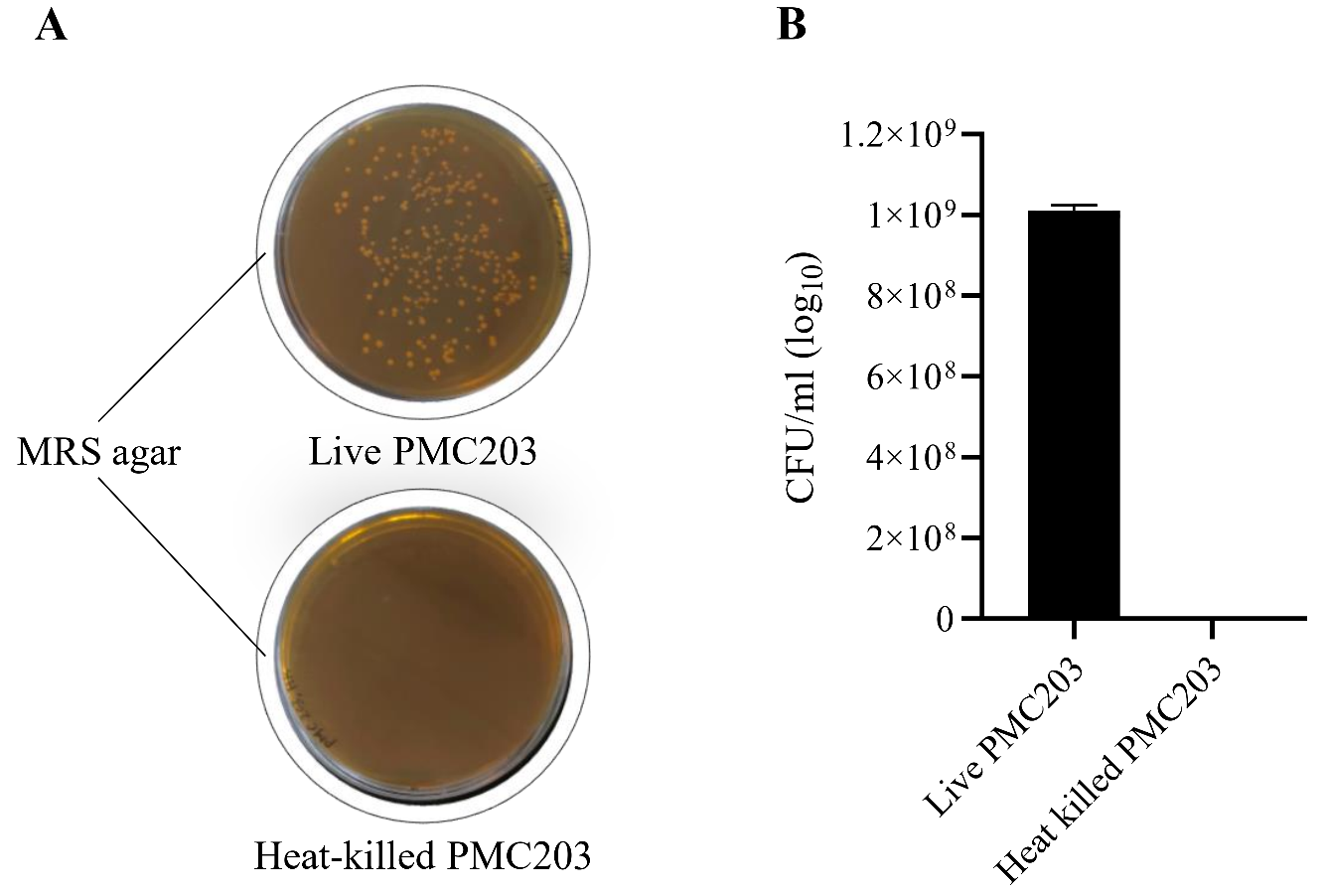


**Fig. S1** Evaluating the inactivation of heat killed PMC203. Live or heat killed PMC203 suspension was inoculated onto the MRS agar and incubated overnight at 37 ℃. (A) Afterward, the plates were checked and (B) CFU was measured indicating the nonviability of heat killed probiotic strain


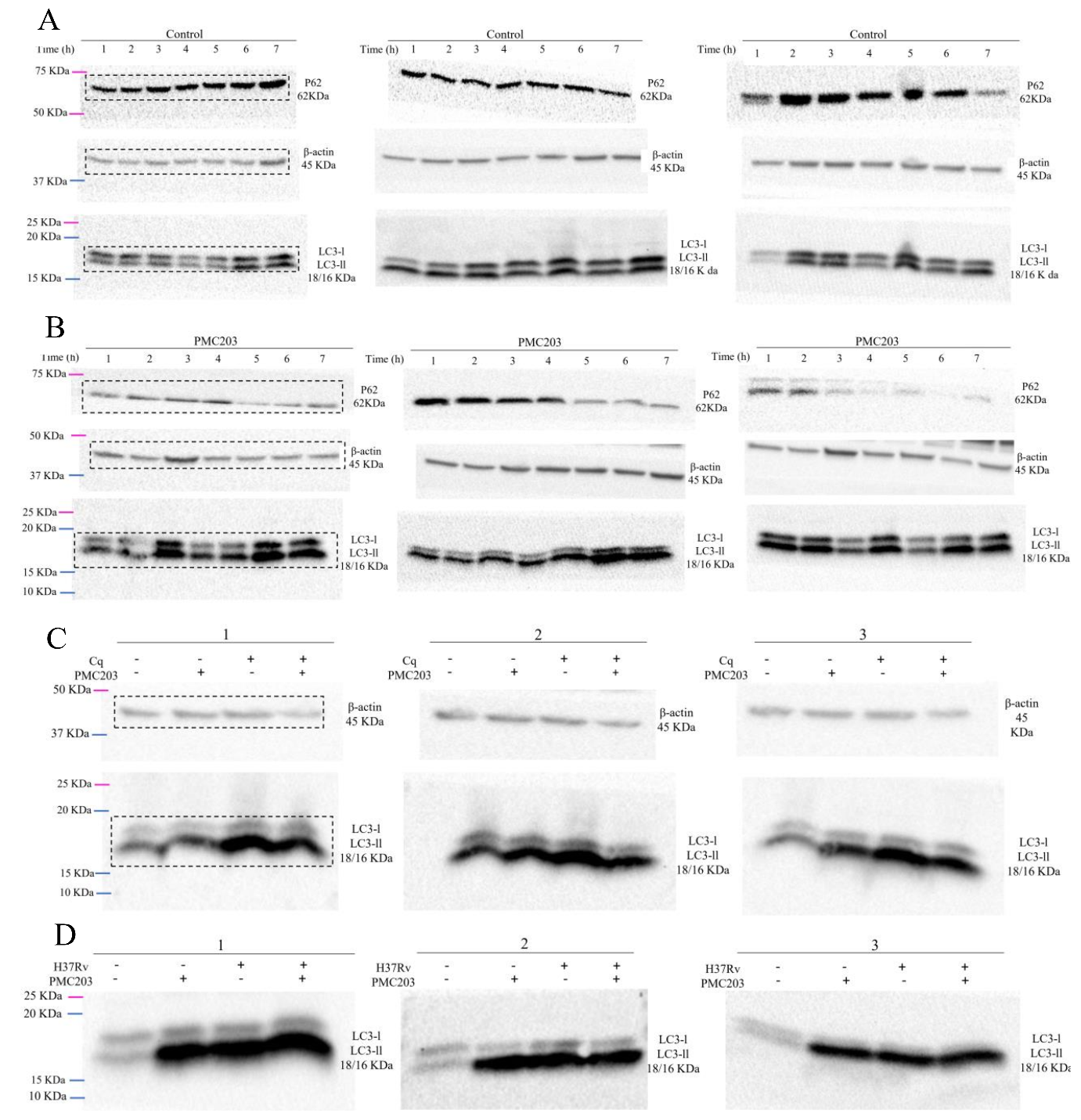


**Fig. S2** Uncropped full-length pictures of western blotting membranes. Triplicate images of (A) control group and (B) PMC treated group, with a selected portion of these pictures presented in the main manuscript as Fig. 2A and Fig. 2B, respectively. (C) Western blot images of Cq conditions, with selected portion presented in the manuscript as Fig. 2G. (D) Lastly, western blot images related to infection conditions. Membranes were often cut to enable blotting for multiple antibodies simultaneously


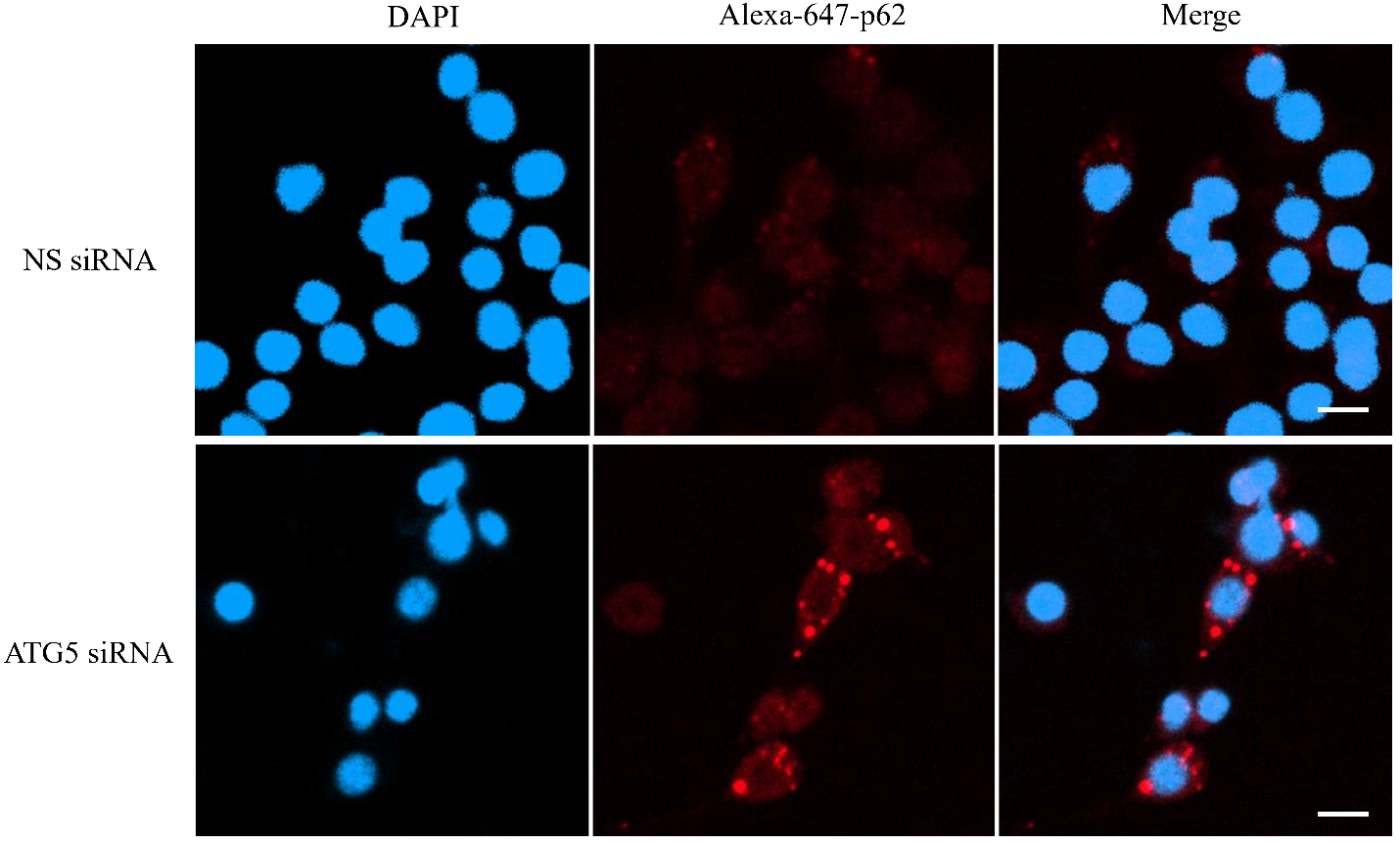


**Fig. S3** Evaluating the ATG5 knockdown efficacy using ATG5 siRNA. The confluent macrophage cells were immunostained with p62 antibody and observed with a confocal microscope. Data showed a visible increase of p62 puncta in the siRNA-treated cells compared to the control. In images, the scale bar indicates a length of 10 μm. The experiment was performed in triplicate, and representative images are shown here.


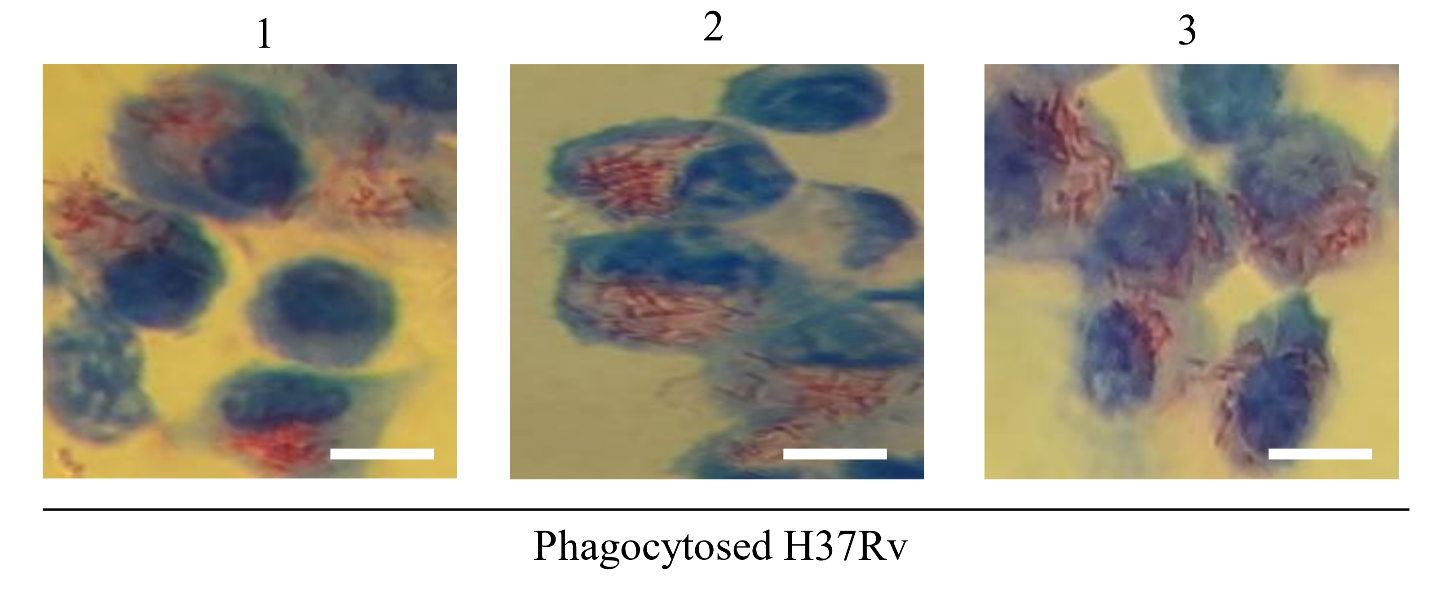


**Fig. S4** Assessment of phagocytosed *M. tuberculosis* in RAW264.7 cells. Confluent monolayer of macrophages was exposed to H37Rv for 2 h. Afterward, cells were washed to remove extracellular H37Rv and acid-fast bacilli staining was conducted, confirming that *M. tuberculosis* was phagocytosed by macrophages in a similar pattern. In images, the scale bar indicates a length of 20 μm. The experiment was performed in triplicate and representative images are shown here.
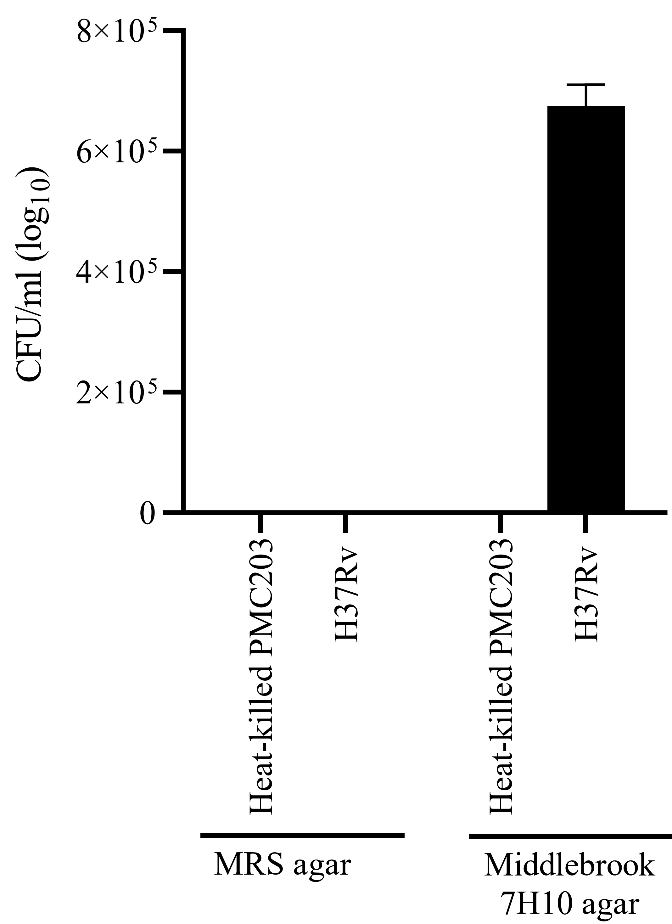


**Fig. S5** Evaluating the growth ability of heat killed PMC203 and H37Rv on MRS and 7H10 medium. A known concentartion of heat killed PMC203 and H37Rv was inoculated onto the MRS and 7H10 agar medium. After incubation period, the plates were checked and showed no growth of heat killed PMC203 and H37Rv onto the MRS agar indicating their inability to grow on this medium. On the other hand, the growth of H37Rv was found onto the 7H10 agar plates that matched the initial known concentration, while no growth of heat-killed PMC203 was observed on the 7H10 medium indicating the ability of *M. tuberculosis* to grow onto the 7H10 agar.


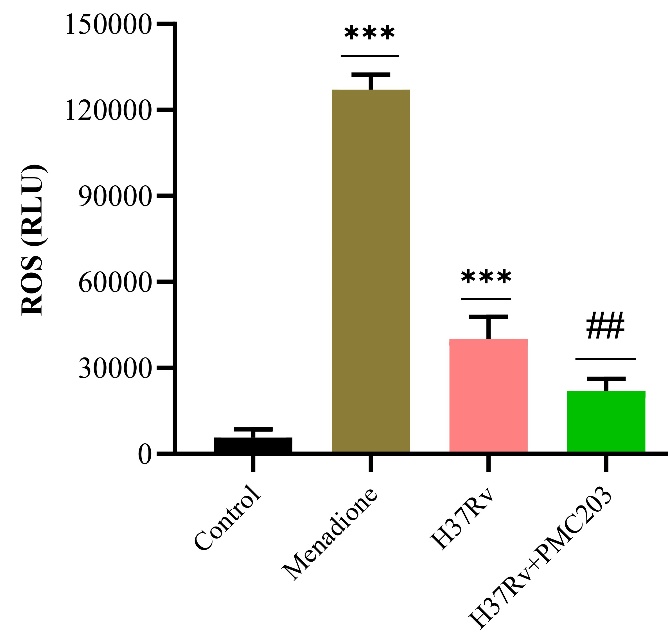


**Fig. S6** Measurement of ROS levels in macrophage cells. Cells were treated with PMC203 and/or H37Rv or menadione (positive control) and fluorescence intensity was measured. Results showed a significant increase of ROS level in cells treated H37Rv alone which further decreased while treating with PMC203.The experiment was performed in triplicate. Results are presented as mean values with corresponding standard deviations. The statistical significance of obtained data was determined using Graph Pad Prism 8.0.1 with a one-way analysis of variance (***, p < 0.001; ##, p < 0.01). *, statistical significance in comparison with the control; #, statistical significance between H37Rv treated group in comparison with H37Rv+PMC203 treated group.

**Supplementary note**

**Supplementary methods and materials for new antituberculosis model**

To investigate the effect of PMC203 stimulated autophagy on reducing *M. tuberculosis* burden, a new model employing a different cell line, J774A.1, was explored in addition to RAW264.7.

**Immunofluorescence assay**

Immunofluorescence staining was conducted to assess the impact of PMC203-mediated autophagy against M*. tuberculosis*. Grown J774A.1 cells in RPMI (Gibco, USA) were seeded on coverslips and subjected to GFP-H37Ra treatment for 2 h at a multiplicity of infection of 30:1 and then washed with PBS for removing extracellular bacteria. Afterward, infected cells were incubated with PMC203 or Rapa for 24 h. Subsequently, cells were washed, fixed, permeabilized, and blocked using PBS, paraformaldehyde, Triton X-100, and bovine serum albumin, respectively. Next, cells were treated with diluted primary LC3 antibody (Sigma, USA) and incubated overnight at 4 ℃. Afterward, cells were incubated with a secondary antibody conjugated with Alexa Fluor 647 (Medchem express, USA) at room temperature for 1 h and finally imaged with a laser-scanning confocal microscope (ZEISS 800 LSM, Germany).

**CFU-based PMC203 mediated anti-tuberculosis assay**

The effectiveness of PMC203-induced autophagy in *M. tuberculosis* killing was also explored employing CFU assay. J774A.1 cells were cultured and exposed to H37Rv for 2 h. Then, they were treated with Cq/ 3-methyladenine (3-MA) for 2 h, washed, and treated with or without PMC203. Finally, cells were lysed at 0, 24, 48, and 72 h of incubation, and diluted lysates were placed onto H710 agar plates for enumerating viable bacteria.


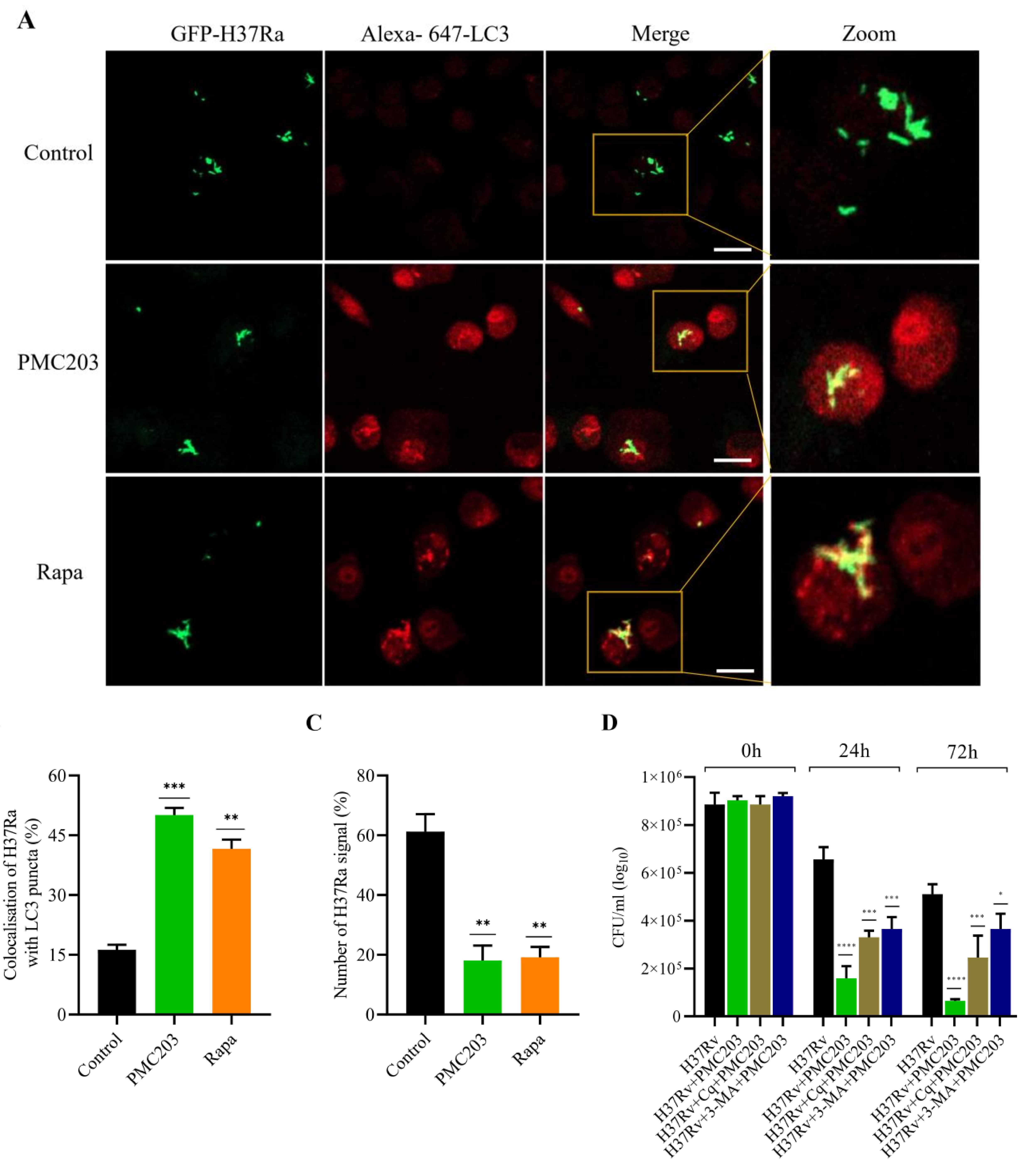


**Fig. S7** Exploring PMC203 induced anti-*M. tuberculosis* effect on J774A.1 cells. Cells were cultured on coverslips, exposed to GFP-H37Ra, and treated with PMC203. (A) After 24 h of incubation, elevated colocalization of GFP-H37Ra with LC3 molecules was noticed in the treated cells. (B) Accordingly, the number of colocalized positive cells were calculated employing image J software. (**C**) The number of positive cells containing > 6 GFP-H37Ra signal was also calculated, in which reduced H37Ra signal was observed in the treated group compared to the untreated group. (D) Additionally, CFU-based anti- *M. tuberculosis* effect was observed utilizing chloroquine (Cq) or 3-methyladenine (3-MA), in which bacterial load was significantly reduced in the PMC203 treated cells compared to untreated cells in a time-dependent manner while their number increased in the Cq/3-MA treated groups. The experiment was performed in triplicate. Results are presented as mean values with corresponding standard deviations. The statistical significance of the obtained data was determined compared to the untreated group using a one-way analysis of variance (*, *p* < 0.05; **, *p* < 0.01; ***, *p* < 0.0001****; *p* < 0.0001)
